# Enantiospecific two-photon electric-dipole selection rule of chiral molecules

**DOI:** 10.1126/sciadv.adz4877

**Published:** 2025-12-03

**Authors:** Fen Zou, Yong Li, Peng Zhang

**Affiliations:** ^1^ Center for Theoretical Physics & School of Physics and Optoelectronic Engineering, Hainan University, Haikou 570228, China.; ^2^ School of Physics, Renmin University of China, Beijing 100872, China.; ^3^ Key Laboratory of Quantum State Construction and Manipulation (Ministry of Education), Renmin University of China, Beijing 100872, China.

## Abstract

Distinguishing between enantiomers is crucial in chemistry and pharmacology. Existing optical methods rely on enantiospecific three-photon electric-dipole transitions, requiring phase locking, three-photon resonance, and precise beam control, limiting their practicality. Here, we propose an optical method that eliminates these constraints by applying a static electric field, which breaks a symmetry combining rotation and time reversal, leading to distinct two-photon selection rules for enantiomers. This enables selective excitation of a target enantiomer using two beams without phase locking or intensity control, greatly improving the feasibility of optical enantiomer differentiation.

## Teaser

An optical method distinguishes enantiomers using a static field and two beams without phase locking or intensity control.

## INTRODUCTION

Chiral molecules are those that have a specific configuration and cannot be superimposed on their mirror images, which are known as enantiomers. The enantiomers with opposite chirality exhibit notable differences in biological activity and chemical behavior (*1*) but have nearly identical physical properties. Efficient approaches of enantiomer differentiation, like enantiodetection (*2*–*16*), enantioseparation (*17*–*20*), and enantiospecific state transfer (*21*–*43*) are crucial in chemical, biological, and pharmaceutical research.

Traditional optical approaches for distinguishing between enantiomers [e.g., various circular dichroisms (*44*, *45*) and Raman optical activity (*46*, *47*)] rely on the weak magnetic-dipole or electric-quadrupole interaction between molecules and microwave (or laser) fields, necessitating high-density samples for detectable signals (*2*). Over the past two decades, numerous optical approaches based on electric-dipole interactions—which are much stronger than magnetic-dipole or electric-quadrupole interaction—have been proposed and experimentally demonstrated (*2*–*43*, *48*). These approaches exploit cyclic electric-dipole transitions among three molecular rovibrational states, driven by three microwave or laser beams. However, these approaches require phase locking, three-photon resonance, and precise control over their intensities and/or operation times. These requirements considerably introduce additional complexity and limit the feasibility.

In this work, we propose an optical method for enantiomer differentiation, which eliminates the necessity for phase locking, resonance condition, and precise control of intensities and operation times of the microwave or laser beams. Our approach uses two microwave or laser beams, as well as a static electric field (E-field). Previous researchers have developed approaches using a weak static E-field and microwave or laser beams to prepare an achiral molecule in a chiral vibrational state (*49*, *50*) or to detect the enantiomeric excess in a mixture of chiral molecules with opposite chirality (*2*). The latter still requires the phase locking and precise control of the intensities of the beams. It was also illustrated that the static E-field can induce enantiospecific sum-frequency generation from optically active liquids (*51*, *52*). Here, we show that, in the presence of a static E-field, the selection rules for certain two-photon electric-dipole transitions between rovibrational states of chiral molecules become enantiospecific because of symmetry changes induced by the static E-field. Specifically, these transitions are driven by two microwave or laser beams linearly polarized in a plane perpendicular to the static E-field. The enantiospecific two-photon selection rule (TPSR) refers to the fact that, when the angle between the polarization directions of these two beams reaches a specific value, the transition is forbidden for one enantiomer but allowed for the other one. Using this enantiospecific TPSR, one can realize enantiospecific transition to distinguish between the enantiomers. Notably, the enantiospecific TPSR is independent of the phases, detunings, intensities, and operation times of the beams. Therefore, precise control or locking of these parameters, as well as resonance conditions, is not necessary for achieving the enantiospecific transition.

## RESULTS

### Two-photon selection rule (TPSR)

We consider a left-handed (right-handed) chiral molecule *L* (*R*) in a static E-field with strength $E=E_{0}e_{z}$ ($E_{0}>0$). Here, $e_{j}$ (j=x,y,z) is the unit vector along the *j* axis of the lab frame. In this work, we assume that $E_{0}$ is weak such that only the **E**-induced coupling between rotational states within the same electronic and vibrational levels needs to be considered. For molecules with dipole moment being on the order of debye and energy gap between vibrational levels being on the order of $\hbar \left(2\pi \right)10^{11}$ Hz or larger, this condition can be safely satisfied when $E_{0}$ is on the order of 10 kV/cm or lower.

We denote the Hamiltonian of the rovibrational states of molecule *s* (s=L,R) as ${\hat{H}}_{0}^{\left(s\right)}\left(E_{0}\right)$. Given that the *z*-component of the total angular momentum of all the nuclei and electrons in the molecule (${\hat{J}}_{z}$) is conserved, the eigenstates and eigenenergies of ${\hat{H}}_{0}^{\left(s\right)}\left(E_{0}\right)$ can be denoted as {∣ξ,M;s〉} and $\left\{\epsilon_{\xi ,M}\right\}$, respectively (see section S2 in the Supplementary Materials). Here, M=0,±1,±2,… is the quantum number of ${\hat{J}}_{z}$. Moreover, ξ=1,2,3,… denotes the energy levels for a fixed *M* and follows the sequence $\epsilon_{1,M}<\epsilon_{2,M}<\epsilon_{3,M}<\ldots$. Given that ${\hat{H}}_{0}^{\left(s\right)}\left(E_{0}\right)$ depends on the strength $E_{0}$ of the static E-field, the eigenstates ∣ξ,M;s〉 and eigenenergies $\epsilon_{\xi ,M}$ are all functions of $E_{0}$. Moreover, $\epsilon_{\xi ,M}$ is independent of the chirality *s* and satisfies $\epsilon_{\xi ,M}=\epsilon_{\xi ,-M}$ because of the time-reversal symmetry. Here, we neglect the tiny parity-violating energy difference because of the fundamental weak force (*53*).

**Fig. 1.**
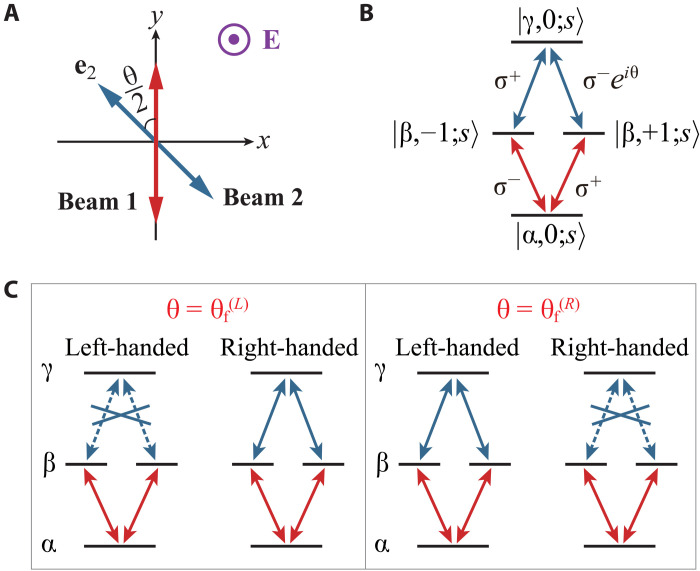
Schematic illustration of enantiospecific TPSR for $\epsilon_{\alpha ,0}<\epsilon_{\beta ,\pm 1}<\epsilon_{\gamma ,0}$. (**A**) Directions of the static E-field **E** (purple) and polarizations of beams 1 (red) and 2 (blue). (**B**) Rovibrational levels and circularly polarized components of beams 1 (red) and 2 (blue) involved in the cascade transition of Eq. 1. (**C**) Enantiospecific transitions: When $\theta =\theta_{f}^{\left(L/R\right)}$, the transition is allowed for the right/left-handed enantiomer but forbidden for the left/right-handed one. (B) and (C) present the schematics for $\epsilon_{\alpha ,0}<\epsilon_{\beta ,\pm 1}<\epsilon_{\gamma ,0}$. The ones for $\epsilon_{\alpha ,0}<\epsilon_{\gamma ,0}<\epsilon_{\beta ,\pm 1}$ are given in fig. S1 of the Supplementary Materials.

Furthermore, two microwave or laser beams, labeled 1 and 2, are applied to the molecule *s*. Both beams are linearly polarized in the *x*-*y* plane. Without the loss of generality, we assume that beam 1 is polarized along $e_{y}$, while beam 2 is polarized along a direction $e_{2}=\text{sin}\left(\theta /2\right)e_{x}-\text{cos}\left(\theta /2\right)e_{y}$ with $\theta \in \left(-\pi ,\pi \right]$ (Fig. 1A). In addition, beam 1 couples with the transition between a rovibrational state ∣α,0;s〉 and two degenerate ones ∣β,±1;s〉, while beam 2 couples with the transition between ∣β,±1;s〉 and another rovibrational state ∣γ,0;s〉. Therefore, the two beams can induce the two-photon cascade transition

∣α,0;s〉→∣β,±1;s〉→∣γ,0;s〉 (1)

and its inverse process for either $\epsilon_{\alpha ,0}<\epsilon_{\beta ,\pm 1}<\epsilon_{\gamma ,0}$ or $\epsilon_{\alpha ,0}<\epsilon_{\gamma ,0}<\epsilon_{\beta ,\pm 1}$.

The above two-photon transitions follow a polarization selection rule, which can be derived as follows. Given that

$$e_{y}\propto \left(e_{+}+e_{-}\right),e_{2}\propto \left(e_{+}+e^{i\theta }e_{-}\right)$$ (2)

with $e_{\pm }\equiv \mp \left(e_{x}\pm ie_{y}\right)/\sqrt{2}$, beam 1 (2) can be decomposed as a superposition of the circularly polarized beams $\sigma^{\pm }$, which are polarized along $e_{\pm }$, with a relative phase 0 (θ). Note that the sign of $e_{\pm }$ defined in this work differs from the one used in some other reference (*54*). Using this result and the selection rules for the electric-dipole couplings induced by circularly polarized electromagnetic waves (Fig. 1B and fig. S1A), we find that beam 1 couples ∣α,0;s〉 only to the superposition state of ∣β,±1;s〉

$$|\psi ;s\rangle =N_{s}^{-1}\left[a_{-}^{\left(s\right)}|\beta ,-1;s\rangle +a_{+}^{\left(s\right)}|\beta ,+1;s\rangle \right]$$ (3)

where

$$a_{\pm }^{\left(s\right)}=\langle \beta ,\pm 1;s|\hat{d}\cdot e_{\pm }|\alpha ,0;s\rangle$$ (4)

with $\hat{d}$ being the molecular electric-dipole operator, and $N_{s}={\left[{\left|a_{-}^{\left(s\right)}\right|}^{2}+{\left|a_{+}^{\left(s\right)}\right|}^{2}\right]}^{1/2}$. Thus, the cascade transition (1) essentially involves only one intermediate state (i.e., ∣ψ;s〉). In addition, the matrix element of the beam 2–induced electric-dipole coupling between ∣ψ;s〉 and ∣γ,0;s〉 satisfies

$$\langle \gamma ,0;s|\hat{d}\cdot e_{2}|\psi ;s\rangle =N_{s}^{-1}\left[a_{-}^{\left(s\right)}b_{+}^{\left(s\right)}+a_{+}^{\left(s\right)}b_{-}^{\left(s\right)}e^{i\theta }\right]$$ (5)

where

$$b_{\pm }^{\left(s\right)}=\langle \gamma ,0;s|\hat{d}\cdot e_{\pm }|\beta ,\mp 1;s\rangle$$ (6)

Moreover, because of the time-reversal symmetry, we have $\left|a_{+}^{\left(s\right)}\right|=\left|a_{-}^{\left(s\right)}\right|$ and $\left|b_{+}^{\left(s\right)}\right|=\left|b_{-}^{\left(s\right)}\right|$. This fact and Eq. 5 yield that the matrix element $\langle \gamma ,0;s|\hat{d}\cdot e_{2}|\psi ;s\rangle$ becomes zero, and thus, the cascade transition (1) is forbidden for the molecule *s* under the condition $\theta =\theta_{f}^{\left(s\right)}\left(E_{0}\right)$, with $\theta_{f}^{\left(s\right)}\left(E_{0}\right)$ being defined as

$$\theta_{f}^{\left(s\right)}\left(E_{0}\right)≔\text{arg}\left[-a_{+}^{\left(s\right)*}\left(E_{0}\right)b_{-}^{\left(s\right)*}\left(E_{0}\right)b_{+}^{\left(s\right)}\left(E_{0}\right)a_{-}^{\left(s\right)}\left(E_{0}\right)\right]$$ (7)

Here, $\text{arg}\left[z\right]\in \left(-\pi ,\pi \right]$ for z∈ℂ. Note that, as mentioned above, the states ∣ξ,M;s〉 (ξ=α,β,γ;M=0,±1;s=L,R) depend on $E_{0}$. Therefore, the coefficients $a_{\pm }^{\left(s\right)}$ and $b_{\pm }^{\left(s\right)}$ defined in Eqs. 4 and 6, as well as the angle $\theta_{f}^{\left(s\right)}$, are functions of $E_{0}$. We call $\theta_{f}^{\left(L/R\right)}\left(E_{0}\right)$ as the forbidden polarization angle of molecule L/R.

Now, we have obtained the TPSR of the cascade transition in Eq. 1. Specifically, this transition is forbidden for the left-handed or right-handed chiral molecule when $\theta =\theta_{f}^{\left(L\right)}\left(E_{0}\right)$ or $\theta =\theta_{f}^{\left(R\right)}\left(E_{0}\right)$, respectively. This TPSR and the expressions of $\theta_{f}^{\left(s\right)}\left(E_{0}\right)$, $a_{\pm }^{\left(s\right)}\left(E_{0}\right)$, and $b_{\pm }^{\left(s\right)}\left(E_{0}\right)$ [i.e., Eqs. 4 to 7] are applicable for both $\epsilon_{\alpha ,0}\epsilon_{\beta ,\pm 1}<\epsilon_{\gamma ,0}$ and $\epsilon_{\alpha ,0}<\epsilon_{\gamma ,0}<\epsilon_{\beta ,\pm 1}$.

We emphasize that the forbidden polarization angles $\theta_{f}^{\left(L,R\right)}\left(E_{0}\right)$ are independent of the phases, detunings, intensities, and operation times of beams 1 and 2. This is because $\theta_{f}^{\left(L,R\right)}\left(E_{0}\right)$ are determined by the factors $a_{\pm }^{\left(L,R\right)}$ and $b_{\pm }^{\left(L,R\right)}$, which, according to Eqs. 4 and 6, are independent of the aforementioned beam parameters.

### Enantiospecific TPSR

Now, there is a natural question: Are the forbidden polarization angles $\theta_{f}^{\left(L\right)}\left(E_{0}\right)$ and $\theta_{f}^{\left(R\right)}\left(E_{0}\right)$ of the left- and right-handed molecules the same or not? It is clear that $\theta_{f}^{\left(L\right)}=\theta_{f}^{\left(R\right)}$ in the absence of the static E-field. However, when the static E-field is applied (i.e., when $E_{0}\neq 0$), we generally find that $\theta_{f}^{\left(L\right)}\neq \theta_{f}^{\left(R\right)}$. In other words, the TPSR becomes enantiospecific.

**Fig. 2.**
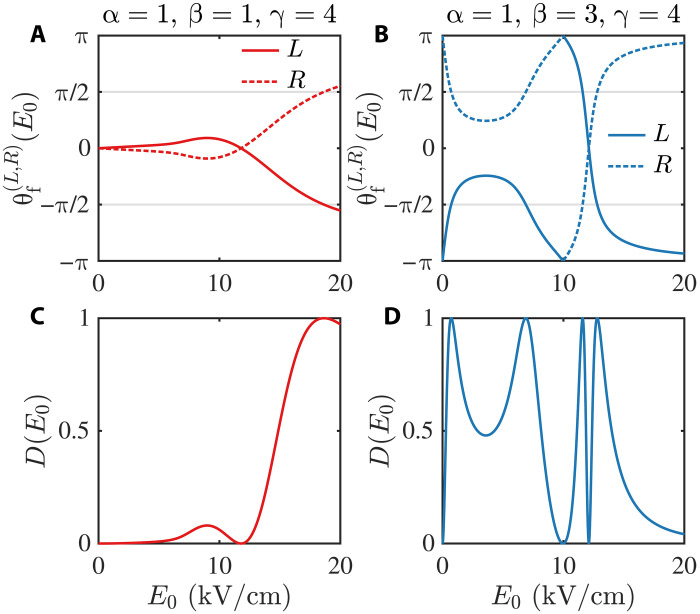
Forbidden polarization angle $\theta_{f}^{\left(L,R\right)}$ and enantiospecificity degree *D* of 1,2-propanediol. (**A** and **B**) Forbidden polarization angle $\theta_{f}^{\left(L,R\right)}\left(E_{0}\right)$. (**C** and **D**) Degree $D\left(E_{0}\right)$ of enantiospecificity of the TPSR. Here, we show the results for transitions of Eq. 1 for 1,2-propanediol molecules, with relevant quantum numbers being (α=1;β=1;γ=4) [(A) and (C)] and (α=1;β=3;γ=4) [(B) and (D)]. In our calculation, we use the rotational constants for a 1,2-propanediol molecule as A=ℏ(2π)8572.05 MHz, B=ℏ(2π)3640.10 MHz, and C=ℏ(2π)2790.96 MHz (*55*). Moreover, in our calculations, we only take into account the rotational states with J≤5, where *J* denotes the quantum number of the total molecular angular momentum. This approximation is sufficiently accurate for our cases with $E_{0}\leq 20\;\text{kV}/\text{cm}$, as shown in section S2 of the Supplementary Materials, where additional calculation details are provided.

Before providing an explanation for this conclusion, we first illustrate it using the results for a typical chiral molecule. We calculate the forbidden polarization angles $\theta_{f}^{\left(L,R\right)}\left(E_{0}\right)$ for a left- or right-handed 1,2-propanediol molecule in the static E-field with $E_{0}\leq 20\;\text{kV}/\text{cm}$, with various specific choices of states ∣α,0,s〉, ∣β,±1,s〉, and ∣γ,0,s〉 (s=L,R), and present the most representative results in Fig. 2 (A and B). The details of the calculation and more results are given in section S2 of the Supplementary Materials. These results indicate that, in general, $\theta_{f}^{\left(L\right)}\left(E_{0}\right)\neq \theta_{f}^{\left(R\right)}\left(E_{0}\right)$ for a nonzero $E_{0}$. In addition, the figures suggest that $\theta_{f}^{\left(L\right)}\left(E_{0}\right)=-\theta_{f}^{\left(R\right)}\left(E_{0}\right)$, as will be demonstrated later.

We further define the forbidden polarization directions $e_{2f}^{\left(s\right)}$ as $e_{2f}^{\left(s\right)}\left(E_{0}\right)≔\text{sin}\left[\theta_{f}^{\left(s\right)}\left(E_{0}\right)/2\right]e_{x}-\text{cos}\left[\theta_{f}^{\left(s\right)}\left(E_{0}\right)/2\right]e_{y}$ for s=L,R. Thus, the TPSR can be reexpressed as follows: The cascade transition (1) is forbidden for molecule *s* when $e_{2}=e_{2f}^{\left(s\right)}$. Therefore, the degree of enantiospecificity of the TPSR can be defined as

$$D≔{\left|e_{2f}^{\left(L\right)}\times e_{2f}^{\left(R\right)}\right|}^{2}={\text{sin}}^{2}\left\{\frac{1}{2}\left[\theta_{f}^{\left(L\right)}-\theta_{f}^{\left(R\right)}\right]\right\}$$ (8)

which satisfies D∈[0,1]. The TPSR is enantiospecific [$e_{2f}^{\left(L\right)}\neq e_{2f}^{\left(R\right)}$] if D≠0. In particular, in the case with *D* = 1 [i.e., ... $e_{2f}^{\left(L\right)}⊥e_{2f}^{\left(R\right)}$], when the cascade transition is forbidden for the left-handed enantiomer (i.e., $\langle \gamma ,0;L|\hat{d}\cdot e_{2}|\psi ;L\rangle =0$), the absolute value of the matrix element $\langle \gamma ,0;R|\hat{d}\cdot e_{2}|\psi ;R\rangle$ of the right-handed enantiomer just reaches its maximum value, and vice versa. In this case, the TPSR is enantiospecific to the maximum extent.

In Fig. 2 (C and D), we illustrate the value of *D* for the 1,2-propanediol molecule as a function of the static E-field strength $E_{0}$. These results, along with those presented in fig. S3 of the Supplementary Materials for additional cases, clearly demonstrate that when $E_{0}$ reaches 1 to 20 kV/cm, situations usually arise where *D* becomes comparable to its maximum value of 1, indicating that the TPSR is highly enantiospecific.

Now, we present the origin of the enantiospecific TPSR through a symmetry analysis. For convenience, we denote *x*, *y*, and *z* as the spatial coordinates of the particles (atomic nuclei and electrons) of each molecule in the center-of-mass (CoM) frame of this molecule. Specifically, $x≔\left\{x_{1},x_{2},\ldots \right\}$, where $x_{i}$ (i=1,2,…) is the *x*-coordinate of particle *i*, and *y* and *z* are defined similarly. Note that the origin of the CoM frame is located at the CoM position of the molecule, and the axes of this frame are parallel to those of the lab frame.

We perform our analysis in the Hilbert space H spanned by both sets of basis of {∣ξ,M;L〉} and {∣ξ,M;R〉}. Every state ∣Ψ〉∈H is a state of the relative motion of these particles and can be described by a wave function 〈x,y,z∣Ψ〉.

Here, we introduce the following transformations:

1) $\hat{P}$: (x,y,z)→(−x,−y,−z) (spatial inversion).

2) ${\hat{C}}_{2x}$: (x,y,z)→(x,−y,−z) (rotation along the *x* axis for π).

3) $\hat{T}$: time reversal.

In space H, the total single-molecule Hamiltonian is ${\hat{H}}_{0}\left(E_{0}\right)≔{\hat{H}}_{0}^{\left(L\right)}\left(E_{0}\right)+{\hat{H}}_{0}^{\left(R\right)}\left(E_{0}\right)$, where ${\hat{H}}_{0}^{\left(L,R\right)}\left(E_{0}\right)$ were introduced before. It can be directly proven that, in the Supplementary Materials, both ${\hat{H}}_{0}\left(E_{0}\right)$ and ${\hat{J}}_{z}$ are invariant under the combined transformation ${\hat{P}\hat{C}}_{2x}\hat{T}$, regardless of whether $E_{0}$ is zero or nonzero. Thus, the states ∣ξ,M;R〉 and ∣ξ,M;L〉, which are degenerate common eigenstates of ${\hat{H}}_{0}$ and ${\hat{J}}_{z}$, can be related via $|\xi ,M;R\rangle ={\hat{P}\hat{C}}_{2x}\hat{T}|\xi ,M;L\rangle$. Using this fact and Eqs. 4 and 6, we obtain $a_{\pm 1}^{\left(R\right)}=-a_{\pm 1}^{{\left(L\right)}^{*}}$ and $b_{\pm 1}^{\left(R\right)}=-b_{\pm 1}^{{\left(L\right)}^{*}}$ (see section S4.3 in the Supplementary Materials). This result and Eq. 7 directly yield that $\theta_{f}^{\left(L\right)}=\theta_{f}^{\left(R\right)}$ only when the parameter $a_{+}^{{\left(s\right)}^{*}}b_{-}^{{\left(s\right)}^{*}}b_{+}^{\left(s\right)}a_{-}^{\left(s\right)}$ is real.

Furthermore, as proven in section S4.4 of the Supplementary Materials, the factor $a_{+}^{\left(s\right)*}b_{-}^{\left(s\right)*}b_{+}^{\left(s\right)}a_{-}^{\left(s\right)}$ (s=L,R) is real, provided that ${\hat{H}}_{0}$ is invariant under the combined transformation ${\hat{C}}_{2x}\hat{T}$. This condition is satisfied for $E_{0}=0$, given that ${\hat{H}}_{0}\left(E_{0}=0\right)$ is invariant under both ${\hat{C}}_{2x}$ and $\hat{T}$. Thus, $\theta_{f}^{\left(R\right)}\left(E_{0}=0\right)=\theta_{f}^{\left(L\right)}\left(E_{0}=0\right)$. When $E_{0}\neq 0$, ${\hat{H}}_{0}\left(E_{0}\right)$ is still invariant under $\hat{T}$, but not invariant under ${\hat{C}}_{2x}$, and thus not invariant under ${\hat{C}}_{2x}\hat{T}$. As a result, the factor $a_{+}^{\left(s\right)*}b_{-}^{\left(s\right)*}b_{+}^{\left(s\right)}a_{-}^{\left(s\right)}$ is generally not real, and thus, we generally have $\theta_{f}^{\left(R\right)}\neq \theta_{f}^{\left(L\right)}$.

Here, we also point out that the aforementioned results $a_{\pm 1}^{\left(R\right)}=-a_{\pm 1}^{{\left(L\right)}^{*}}$ and $b_{\pm 1}^{\left(R\right)}=-b_{\pm 1}^{{\left(L\right)}^{*}}$ and Eq. 7 also yield $\theta_{f}^{\left(R\right)}=-\theta_{f}^{\left(L\right)}$ (mod 2π). Thus, the degree *D* of enantiospecificity of the TPSR takes its maximum value (i.e., *D* = 1) only when $\left|\theta_{f}^{\left(L,R\right)}\right|=\pi /2$ and becomes zero only when $\theta_{f}^{\left(L,R\right)}=0$ or π.

Above is the symmetry analysis of the origin of the enantiospecific TPSR. In addition to this analysis, we can also understand this TPSR through a simpler, more intuitive picture, which is presented in section S4.5 of the Supplementary Materials.

### Enantiospecific transitions

With the help of the enantiospecific TPSR, one can realize enantiospecific transitions, i.e., the transition from α to γ, which occurs exclusively for the enantiomer with a specific chirality. For instance, when the angle θ of the polarization direction of beam 2 is tuned to $\theta_{f}^{\left(L\right)}$, the left-handed molecules can only be transferred from α to β by beam 1 and cannot be further transferred from β to γ by beam 2. In contrast, the right-handed molecules can be transferred from level α to β and subsequently to γ by the two beams [Fig. 1C and fig. S1B of the Supplementary Materials, left]. Therefore, if a molecule is detected at the γ level, it is guaranteed to be right-handed. Similarly, another enantiospecific transition can be realized when θ is tuned to $\theta_{f}^{\left(R\right)}$ [Fig. 1C and fig. S1B of the Supplementary Materials, right]. Note that these results are independent of the detunings, phases, intensities, and operational times of the two beams. As a result, the resonance condition and precise control or locking of these parameters are not required for the realization of enantiospecific transitions.

**Fig. 3.**
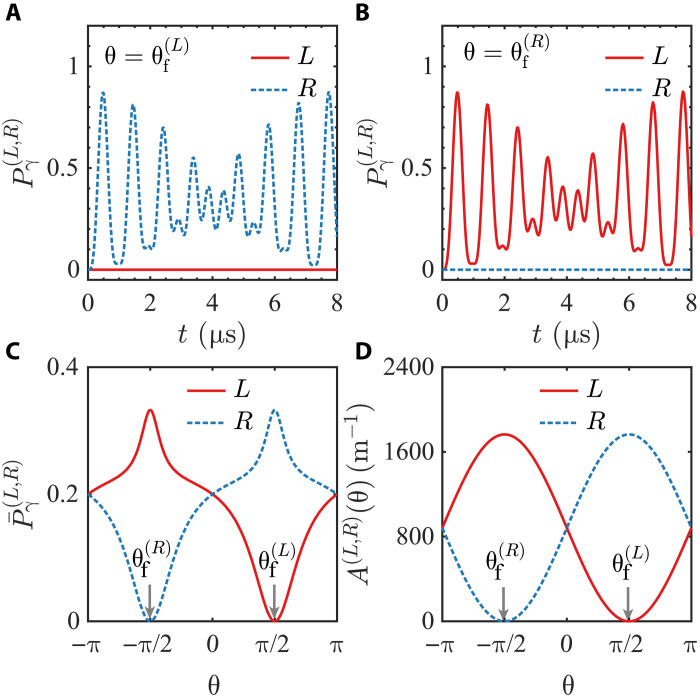
Final-state probability and beam-absorption of molecules with enantiospecific transitions. (**A** and **B**) Time evolution of the final-state probability $P_{\gamma }^{\left(L,R\right)}\left(t\right)$ of the cascade transition (1) for $\theta =\theta_{f}^{\left(L\right)}$ (A) and $\theta =\theta_{f}^{\left(R\right)}$ (B). (**C**) Time-averaged final-state probability ${\bar{P}}_{\gamma }^{\left(L,R\right)}$ as a function of θ. In (A) to (C), we consider the cases with $\theta_{f}^{\left(L\right)}=\pi /2$, $\theta_{f}^{\left(R\right)}=-\pi /2$, $\epsilon_{\beta ,\pm 1}-\epsilon_{\alpha ,0}-\omega_{1}=\left(2\pi \right)\;0.1$ MHz, $\epsilon_{\gamma ,0}-\epsilon_{\beta ,\pm 1}-\omega_{2}=\left(2\pi \right)\;0.4$ MHz, and $\Omega_{1}=\Omega_{2}=\left(2\pi \right)\;1$ MHz, with $\omega_{1\left(2\right)}$ and $\Omega_{1\left(2\right)}$ being the angular frequency of beam 1 (2) and the Rabi frequency of the transition induced by beam 1 (2), respectively. (**D**) Absorption $A^{\left(L,R\right)}\left(\theta \right)$ of beam 2 of left- and right-handed enantiomers. The meaning of the absorption A(θ) is as follows: When the incident intensity $I_{0}$ of beam 2 is weak, the intensity of this beam decays with the propagation distance r in the molecules as $I_{0}\text{exp}\left[-A\left(\theta \right)r\right]$. In (D), we show the results for the system with molecular number density being $3.22\times 10^{18}\;m^{-3}$, the angular frequency of beam 2 being $\left(2\pi \right)\;6\times 10^{14}\;\text{Hz}$, the spontaneous emission rates from ∣γ,0,s〉 to the lower states ∣α,0;s〉 and ∣β,±1;s〉, as well as those from ∣β,±1,s〉 to ∣α,0,s〉, all being (2π) 10 MHz, and the electric dipole moment with respect to the β→γ transition being 3 D. Other parameters used in the calculation are the same as (C). The details of the calculations for (A) to (D) are given in section S5 of the Supplementary Materials, where the essential role of the degeneracy of the β-states ∣β,±1;s〉 for the emergence of the TPSR is also clarified with these calculations.

We illustrate the enantiospecific transition by solving the time-dependent Schrödinger equation of the rovibrational state $|\Psi^{\left(s\right)}\left(t\right)\rangle$ of molecule *s* (s=L,R) for the transition (1), with the forbidden polarization angles being $\theta_{f}^{\left(L/R\right)}=\pm \pi /2$ and the initial state being (see section S5.1 in the Supplementary Materials) $|\Psi^{\left(s\right)}\left(0\right)\rangle =|\alpha ,0;s\rangle$. We show the time evolution of the final-state probability $P_{\gamma }^{\left(s\right)}\left(t\right)={|\langle \gamma ,0;s|\Psi^{\left(s\right)}\left(t\right)\rangle |}^{2}$ (s=L,R) for the cases with $\theta =\theta_{f}^{\left(L\right)}$ and $\theta =\theta_{f}^{\left(R\right)}$ in Fig. 3 (A and B), respectively. As shown in Fig. 3A, when $\theta =\theta_{f}^{\left(L\right)}$, the probability $P_{\gamma }^{\left(L\right)}\left(t\right)$ is always zero, while $P_{\gamma }^{\left(R\right)}\left(t\right)$ oscillates with time. In other words, the two-photon transition is forbidden for the left-handed enantiomer but allowed for the right-handed one. When $\theta =\theta_{f}^{\left(R\right)}$, there is a similar result, as shown in Fig. 3B. A comparison between these results and those obtained using three-microwave approaches is given in section S6 of the Supplementary Materials.

Moreover, when θ is not tuned to $\theta_{f}^{\left(L\right)}$ or $\theta_{f}^{\left(R\right)}$, the α↔γ transition can occur for both enantiomers and thus is not enantiospecific. However, given that the matrix elements $\langle \gamma ,0;s|\hat{d}\cdot e_{2}|\psi ;s\rangle$ for s=L and s=R are unequal, there are still a quantitative difference between the final-state probabilities ${\bar{P}}_{\gamma }^{\left(L,R\right)}\left(t\right)$ of enantiomers with opposite chirality. We illustrate this in Fig. 3C, where the time-averaged final-state probability ${\bar{P}}_{\gamma }^{\left(L,R\right)}≔{\text{lim}}_{T\to \infty }\frac{1}{T}\int_{0}^{T}P_{\gamma }^{\left(L,R\right)}\left(t\right)\mathrm{dt}$ is plotted as a function of θ, for the above example.

The above results can be experimentally observed through the direct measurement of the molecular population $P_{\gamma }^{\left(L,R\right)}$ using methods such as those described in refs. (*37*, *40*, *43*). In addition, the enantiospecific transitions can also be verified by detecting either the absorption of beam 2 by the molecules or the fluorescence (or microwave) emitted from molecules excited to the γ-state by beam 2. The molecules considered here have chirality *s* (s=L/R, denoting left- and right-handed chirality). They are initially prepared in the state ∣α,0;s〉 and interact with beam 1 and the static E-field. We denote the absorption rate or fluorescence (or microwave) intensity as $A^{\left(s\right)}\left(\theta \right)$, a function of the polarization angle θ of beam 2. Because of the enantiospecific transition, when $\theta =\theta_{f}^{\left(L\right)}$, we have $A^{\left(L\right)}=0$ and $A^{\left(R\right)}\neq 0$; conversely, when $\theta =\theta_{f}^{\left(R\right)}$, we have $A^{\left(R\right)}=0$ and $A^{\left(L\right)}\neq 0$. For other values of θ, $A^{\left(L,R\right)}$ of the two enantiomers are both nonzero but unequal. These results are illustrated in Fig. 3D, where the absorption rate of beam 2 is plotted as a function of θ for a representative example. Further discussions on the experimental feasibility of realizing TPSR and observing the enantiospecific transition effects are provided in sections S7 and S8 of the Supplementary Materials, respectively.

## DISCUSSION

### Applications

The enantiospecific transitions based on enantiospecific TPSR can be applied to enantiospecific state transfer, enantiodetection, and enantioseparation. For instance, in a mixture of enantiomers, the absorption rate of beam 2 or the intensity of fluorescence (or microwave radiation) emitted from molecules excited to the γ-state by beam 2—both denoted as A(θ)—satisfies

$$\frac{A\left[\theta_{f}^{\left(R\right)}\right]}{A\left[\theta_{f}^{\left(L\right)}\right]}=\frac{n^{\left(L\right)}}{n^{\left(R\right)}}$$ (9)

provided that the weak beam 2 is weak enough so that the γ-state is far from saturation and that the propagation distances of beam 2 in the left- and right-handed molecules are the same (see section S5.2 in the Supplementary Materials). Here, $n^{\left(L\right)}$ and $n^{\left(R\right)}$ represent the densities of the left- and right-handed enantiomers, respectively. Thus, the number ratio of molecules with different chirality, which equals to $n^{\left(L\right)}/n^{\left(R\right)}$, can be determined by measuring $A\left[\theta_{f}^{\left(L\right)}\right]$ and $A\left[\theta_{f}^{\left(R\right)}\right]$.

In addition, to realize enantioseparation, one can exclusively transfer the molecules with a certain chirality to the γ level and then ionize or dissociate the molecules in the γ level via a laser. By recombining these ionized or dissociated products, one can obtain molecules with the certain chirality.

As mentioned earlier, the resonance condition and phase locking of the two beams, as well as precise control over their intensities and operation times, are not required to realize enantiospecific transitions. These factors simplify the procedure and enhance the feasibility of the aforementioned applications. In particular, the frequencies of the two beams can be chosen independently and arbitrarily, and the energy levels α, β, and γ can be selected freely, either within the same electronic or vibrational state, or not. Consequently, the intermediate state β and the final state γ can be chosen to be sufficiently high such that thermal occupation at room temperature in these states can be safely neglected. As a result, the enantiospecific transitions can be achieved at room temperature, in contrast to the current experimental demonstrations (*2*, *3*, *37*, *40*, *41*, *43*) of enantiomer differentiation methods based on three-level cyclic transitions, which currently require temperatures on the order of a few kelvins and the use of depletion techniques (*40*, *43*) to mitigate the adverse effects of thermal population in rotational states.

The enantiospecific transitions occur only when θ is tuned to $\theta_{f}^{\left(L\right)}$ or $\theta_{f}^{\left(R\right)}$. However, as shown in Fig. 3 (C and D), when θ takes other values, the α↔γ transition for enantiomers with different chirality remains quantitatively different. These distinct behaviors can also be used for applications such as enantiodetection and enantioseparation. In such cases, controlling beam detunings (equivalently, frequencies), intensities, or operation times may be necessary, while phase locking is generally still not required. Thus, the advantage of not needing phase locking remains intact.

## Supplementary Materials

### This PDF file includes:

Sections S1 to S8 Figs. S1 to S8 References
