# Supplementary material for: Enantiospecific two-photon electric-dipole selection rule of chiral molecules
Source: Sci Adv. 2025 Dec 3;11(49):eadz4877. doi: 10.1126/sciadv.adz4877 (PMC13142755; doi:10.1126/sciadv.adz4877)
Supplement: Supplementary file 1 — Sections S1 to S8 Figs. S1 to S8 References [file sciadv.adz4877_sm.pdf]

Science Advances
AAAS

Supplementary Materials for

Enantiospecific two-photon electric-dipole selection rule of chiral molecules

Fen Zou *et al.*

Corresponding author: Yong Li, yongli@hainanu.edu.cn; Peng Zhang, pengzhang@ruc.edu.cn

Sci. Adv. **11**, eadz4877 (2025)
DOI: 10.1126/sciadv.adz4877

This PDF file includes:

Sections S1 to S8
Figs. S1 to S8
References

S1 Schematics for $\epsilon_{\alpha,0} < \epsilon_{\gamma,0} < \epsilon_{\beta,\pm1}$.

In Fig. 1(B and C) of the main text, we present the schematics for the two-photon cascade transition of Eq. (1) and the TPSR for the cases with $\epsilon_{\alpha,0} < \epsilon_{\beta,\pm1} < \epsilon_{\gamma,0}$. Here we give the schematics for another kind of cases, i.e., the ones with $\epsilon_{\alpha,0} < \epsilon_{\gamma,0} < \epsilon_{\beta,\pm1}$, in Fig. S1.

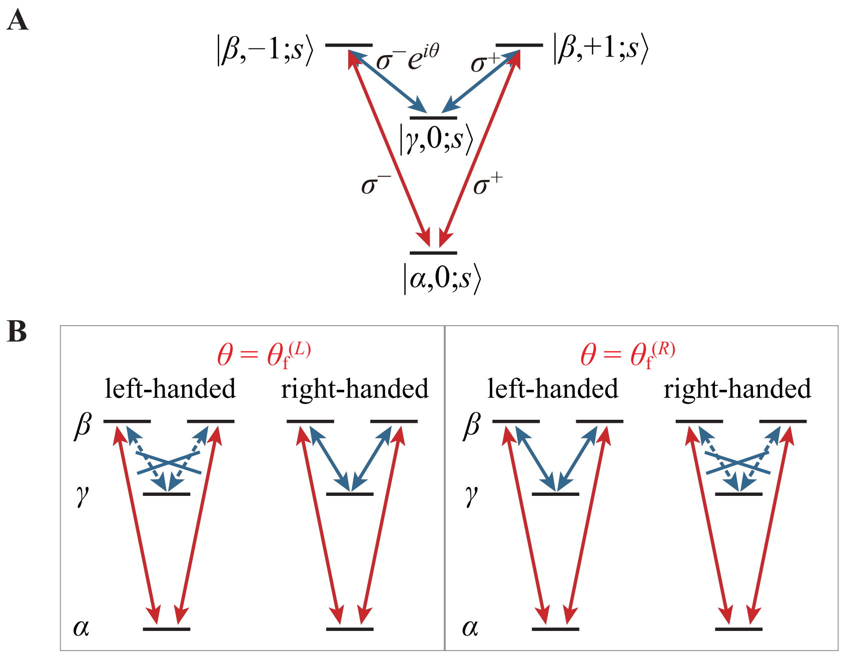

Figure S1: **Schematic illustration of enantiospecific TPSR for** $\epsilon_{\alpha,0} < \epsilon_{\gamma,0} < \epsilon_{\beta,\pm1}$. (**A**) The rovibrational levels and the circularly-polarized components of beams 1 (red) and 2 (blue) involved in the cascade transition of Eq. (1). (**B**) Enantiospecific transitions.

S2 Calculation for Results of Fig. 2

In this section we show our approach for the calculation of the forbidden polarization angle $\theta_f^{(L,R)}(E_0)$ of the left- and right-handed 1,2-propanediol molecules, i.e., the results of Fig. 2 in the main text. We first derive the results for the left-handed molecule, and then derive the results for the right-handed one.

S2.1 The Left-Handed Molecule ($s = L$)

We first derive the eigen-states $|\xi,M;L\rangle$ ($\xi = 1, 2, ...; M = 0, \pm 1, ...$) and the corresponding eigen-energies $\epsilon_{\xi,M}$ of the Hamiltonian $\hat{H}_0^{(L)}(E_0)$ of the left-handed molecule L in the static E-field $\boldsymbol{E} = E_0\mathbf{e}_z$. This Hamiltonian can be expressed as

$$\hat{H}_0^{(L)}(E_0) = \hat{H}_{\rm F}^{(L)} - E_0\hat{\boldsymbol{d}} \cdot \mathbf{e}_z, \tag{S1}$$

where $\hat{H}_{\rm F}^{(L)}$ is the Hamiltonian in the absence of the static E-field, and $\hat{\boldsymbol{d}}$ is the molecular electric-dipole operator.

As mentioned in the main text, in this work we assume E_0 is weak enough so that we only require to consider the E_0-induced coupling between the eigen-states of $\hat{H}_{\rm F}^{(L)}$ (i.e., the rotational states) in the same electronic and vibrational level. On the other hand, as shown in the caption of Fig. 2, we focus on several lowest eigen-states of $\hat{H}_0^{(L)}(E_0)$ (i.e., the states with quantum numbers $\alpha, \beta, \gamma < 4$). Due to these facts, in the calculation we only consider the rotational states in the lowest electronic and vibrational level.

For the convenience of the following calculation, we denote the three principal axes of inertia of the left-handed molecule as a, b, and c, with corresponding moments of inertia I_a, I_b, and I_c. Without loss of generality, we assume $I_a < \{I_b, I_c\}$, and the axes a, b, and c form a right-handed frame. We further introduce the symmetric-top basis $\{|J, K, M; L\rangle\}$ of the rotational states of the lowest electronic and vibrational level. Here $J = 0, 1, 2, ...$ is the quantum number of $\hat{\boldsymbol{J}}^2$, with $\hat{\boldsymbol{J}}$ being the total angular momentum, and M and K are quantum numbers of of the angular momentum along the z-axis of the lab frame (i.e., $\hat{J}_z$) and the one along the principal axis a (i.e., $\hat{J}_a$), respectively.

To derive the eigen-states and eigen-values of $\hat{H}_0^{(L)}(E_0)$, we express the terms in the right-hand-side of Eq. (S1) as matrices in the basis $\{|J, K, M; L\rangle\}$. The matrix elements of $\hat{H}_{\rm F}^{(L)}$ in this basis are given by (*54*)

$$\langle J, K, M; L|\hat{H}_{\rm F}^{(L)}|J', K', M'; L\rangle = \delta_{J,J'}\delta_{M,M'}\left[f(J, K)\delta_{K',K} + g_{\pm}(J, K)\delta_{K',K\pm 2}\right]; \tag{S2}$$

where $\delta_{\alpha,\beta}$ is the Kronecker delta symbol, and the functions $f(J,K)$ and $g^{(\pm)}(J,K)$ are defined as

$$f(J,K) = \frac{1}{2}(B+C)\left[J(J+1)-K^2\right]+AK^2; \tag{S3}$$

$$g_\pm(J,K) = \frac{1}{4}(B-C)\left\{\left[J(J+1)-K(K\pm1)\right]\left[J(J+1)-(K\pm1)(K\pm2)\right]\right\}^{1/2}, \tag{S4}$$

with

$$A=\frac{\hbar^2}{2I_a},\ B=\frac{\hbar^2}{2I_b},\ C=\frac{\hbar^2}{2I_c}. \tag{S5}$$

For a 1,2-propanediol molecule, we have $A=\hbar(2\pi)8572.05\,\mathrm{MHz}$, $B=\hbar(2\pi)3640.10\,\mathrm{MHz}$, and $C=\hbar(2\pi)2790.96\,\mathrm{MHz}$ (*55*). Additionally, the matrix element of the term $\hat{\boldsymbol{d}}\cdot\mathbf{e}_z$ in the basis $\{|J,K,M;L\rangle\}$ can be expressed as (*56, 57*)

$$\begin{aligned}&\langle J,K,M;L|(\hat{\boldsymbol{d}}\cdot\mathbf{e}_z)|J',K',M';L\rangle\\ &= \delta_{M,M'}\sqrt{(2J+1)(2J'+1)}\begin{pmatrix} J & 1 & J' \\ M & 0 & -M'\end{pmatrix}\left\{\sum_{\sigma'=0,\pm1}\mu_{\sigma'}^{(m)}(-1)^{M'+1-K'+\sigma'}\begin{pmatrix} J & 1 & J' \\ K & -\sigma' & -K'\end{pmatrix}\right\},\end{aligned} \tag{S6}$$

with $\mu_{0,\pm1}^{(m)}$ being given by

$$\mu_0^{(m)}=-d_a;\quad \mu_\pm^{(m)}=\pm(d_b\pm id_c)/\sqrt{2}, \tag{S7}$$

where (d_a,d_b,d_c) are components of the electric-dipole moment of the molecule L in the principle-axis frame (abc-frame), which are independent of (K,K',M,M'). For a left-handed 1,2-propanediol molecule, we have $d_a=-1.201$ Debye, $d_b=-1.916$ Debye, and $d_c=-0.365$ Debye (*55*).

Notice that Eq. (S6) relates the component of the molecular electric dipole moment in the lab frame ($\hat{\boldsymbol{d}}\cdot\mathbf{e}_z$) to those in the principal-axis frame ($d_{a,b,c}$). In deriving this equation, we have used the wave function for the states $\{|J,K,M;L\rangle\}$ [Eq. (3.125) of Ref. (*54*)], the transformation between the components of a vector in the lab frame and the principal-axis frame [Eq. (3.103) of Ref. (*54*)], and the integral properties of the Wigner-D functions [Eq. (3.118) of Ref. (*54*)]. Specifically, the factors $\sqrt{(2J+1)(2J'+1)}$ and $(-1)^{M'+1-K'+\sigma'}$ in Eq. (S6) come from Eq. (3.125) of Ref. (*54*). Note that in Ref. (*54*) the axes of the lab frame and the principle-axis frame are denoted as (X,Y,Z)-axes and

(x, y, z)-axes, respectively, while in our work they are denoted as (x, y, z)-axes and (a, b, c)-axes, respectively. Furthermore, Eq. (S6) shows that the electric field along the z-direction of the lab frame cannot couple the states $|J, K, M; L\rangle$ and $|J', K', M'; L\rangle$ when $M \neq M'$, due to the rotational symmetry along the z-axis of the lab frame.

Using Eqs. (S2, S6) and Eq. (S1), we can express $\hat{H}_0^{(L)}(E_0)$ as a matrix in the basis $\{|J, K, M; L\rangle\}$. We derive the eigen-states $\{|\xi,M;L\rangle\}$ and eigen-energies $\{\epsilon_{\xi,M}\}$ by directly diagonalizing this matrix. Specifically, the eigen-states $\{|\xi,M;L\rangle\}$ can be expressed as

$$|\xi,M;L\rangle = \sum_{J=|M|}^{+\infty} \sum_{K=-J}^{+J} S_{\xi,J,K;M}^{(L)} |J, K, M; L\rangle, \tag{S8}$$

while the coefficients $\{S_{\xi,J,K;M}^{(L)}\}$ are given by numerical diagonalization of the matrix.

According to Eq. (S8), $P_J(\xi, M; L) := \sum_{K=-J}^{J} |S_{\xi,J,K;M}^{(L)}|^2$ represents the probability of the state $|\xi,M;L\rangle$ within the subspace corresponding to a specific angular-momentum quantum number J. Although P_J is nonzero for each J, it becomes negligible for sufficiently large J. Specifically, in the systems shown in Fig. 2, we focus on the several lowest eigenstates $|\xi,M;L\rangle$ of $\hat{H}_F^{(L)}$. For the static E-field considered in Fig. 2 (at most 20 kV/cm), the probabilities of these states within the subspace with $J > 5$ (i.e., $\sum_{J>5} P_J$) are negligible. To demonstrate this, we present the probability P_J of the states involved in Fig. 2 for the case with $E_0 = 20$ kV/cm in Fig. S2. It is observed that for large $J \geq 3$, P_J almost exponentially decays with J, and as J increases to 5, P_J decreases to at most 10^{-4}. Therefore, the order of magnitude of the probability $\sum_{J>5} P_J$ is estimated to be on the order of 10^{-4}. A direct analysis based on this estimation further reveals that the relative error of $\theta_f^{(L)}$, which is introduced by neglecting the components of these states with $J > 5$, is also on the order of 10^{-4}. Consequently, in our calculations, the summation over J in Eq. (S8) is performed for $J \leq 5$. Additionally, for $J \leq 5$, the energy associated with centrifugal distortion is only of the order of $\hbar(2\pi)$ MHz (*58*), which is significantly smaller than the gaps between the eigenenergies of $\hat{H}_F^{(L)}$ (on the order of $\hbar(2\pi)$ GHz). Therefore, we neglect the centrifugal distortion effect in this work. Our estimation shows that this effect becomes non-negligible only when E_0 is at least on the order of 10^4 kV/cm.

Substituting the states $|\xi,M;L\rangle$ of Eq. (S8), which are derived via the above approach, into

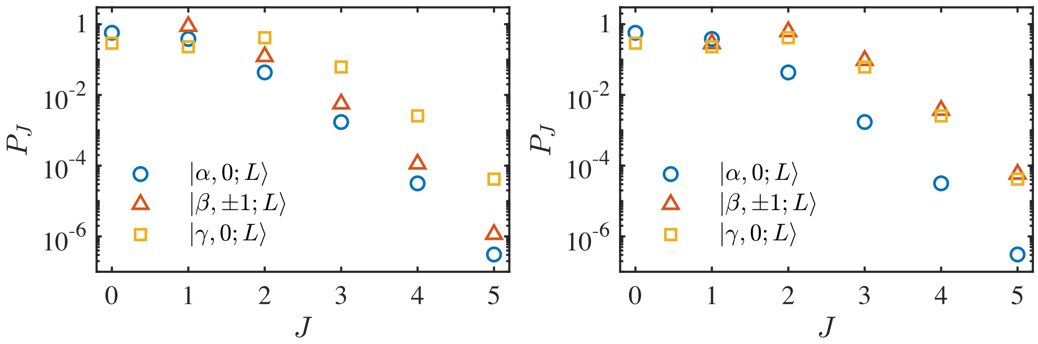

Figure S2: **The probability P_J of states involved in the example of Fig. 2**. Here we show P_J of the states $|\alpha,0;L\rangle$ (blue circle), $|\beta,\pm1;L\rangle$ (red triangle), and $|\gamma,0;L\rangle$ (orange square) in the examples in Fig. 2A (left) and Fig. 2B (right), for $E_0 = 20\,\mathrm{kV/cm}$.

Eqs. (4, 6) of the main text, and using the relations (*56, 57*)

$$\langle J,K,M;L|\big(\hat{\boldsymbol{d}}\cdot\mathbf{e}_\pm\big)|J',K',M';L\rangle$$

$$= \delta_{M,M'\pm1}\sqrt{(2J+1)(2J'+1)}\begin{pmatrix} J & 1 & J' \\ M & \mp1 & -M' \end{pmatrix}\left\{\sum_{\sigma'=0,\pm1}\mu_{\sigma'}^{(m)}(-1)^{M'-K'+\sigma'}\begin{pmatrix} J & 1 & J' \\ K & -\sigma' & -K' \end{pmatrix}\right\}, \tag{S9}$$

we directly obtain the coefficients $a_\pm^{(L)}$ and $b_\pm^{(L)}$. Note that Eq. (S9) can be derived using the same method as Eq. (S6).

Finally, we substitute the coefficients $a_\pm^{(L)}$ and $b_\pm^{(L)}$ derived above into Eq. (7), and obtain the forbidden polarization angle $\theta_{\mathrm{f}}^{(L)}(E_0)$. We also point out that in our derivation we assume the electronic wave function is real. Due to this assumption and the time-reversal symmetry, we have $|a_+^{(L)}| = |a_-^{(L)}|$ and $|b_+^{(L)}| = |b_-^{(L)}|$, as mentioned in our main text.

S2.2 The Right-Handed Molecule ($s = R$)

Now we consider the right-handed 1,2-propanediol molecule. We derive the forbidden polarization angle $\theta_{\mathrm{f}}^{(R)}(E_0)$ of this molecule with the same method as above.

To this end, we introduce the rotational states $\{|J,K,M;R\rangle\}$ of the right-handed molecule R in

the lowest electric and vibrational level, which can be defined as

$$|J, K, M; R\rangle = \hat{\mathcal{P}}|J, K, M; L\rangle, \tag{S10}$$

where $\hat{\mathcal{P}}$ is the spatial inversion operator for the coordinates of all nuclei and electrons in the molecule.

Furthermore, similar as in the above subsection, we express the Hamiltonian $\hat{H}_0^{(R)}(E_0) = \hat{H}_{\mathrm{F}}^{(R)} - E_0\hat{\boldsymbol{d}} \cdot \mathbf{e}_z$ and the operators $\hat{\boldsymbol{d}} \cdot \mathbf{e}_\pm$ as matrices in the basis $\{|J, K, M; R\rangle\}$. Note that since $\hat{\mathcal{P}}\hat{H}_{\mathrm{F}}^{(R)}\hat{\mathcal{P}} = \hat{H}_{\mathrm{F}}^{(L)}$ and $\hat{\mathcal{P}}(\hat{\boldsymbol{d}} \cdot \mathbf{e}_{z,\pm})\hat{\mathcal{P}} = -\hat{\boldsymbol{d}} \cdot \mathbf{e}_{z,\pm}$, we have

$$\langle J, K, M; R|\hat{H}_{\mathrm{F}}^{(R)}|J', K', M'; R\rangle = +\langle J, K, M; L|\hat{H}_{\mathrm{F}}^{(L)}|J', K', M'; L\rangle, \tag{S11}$$

$$\langle J, K, M; R|\big(\hat{\boldsymbol{d}} \cdot \mathbf{e}_{z,\pm}\big)|J', K', M'; R\rangle = -\langle J, K, M; L|\big(\hat{\boldsymbol{d}} \cdot \mathbf{e}_{z,\pm}\big)|J', K', M'; L\rangle. \tag{S12}$$

We calculate the matrix elements for the right-handed molecule by substituting the results of Eqs. (S2, S6, S9) into Eqs. (S11, S12).

After expressing $\hat{H}_0^{(R)}(E_0)$ and $\hat{\boldsymbol{d}} \cdot \mathbf{e}_\pm$ as matrices in the basis $\{|J, K, M; R\rangle\}$, we calculate the eigen-states $\{|\xi, M; R\rangle\}$ and the eigen-energies $\{\epsilon_{\xi,M}\}$ of $\hat{H}_0^{(R)}(E_0)$, and then derive the parameters $a_\pm^{(R)}$, $b_\pm^{(R)}$, and the forbidden polarization angle $\theta_{\mathrm{f}}^{(R)}(E_0)$, with the same method as in the above subsection.

S3 $\theta_{\mathrm{f}}^{(L,R)}(E_0)$ and $D(E_0)$ for More Cases

In Fig. 2 of our main text we show the forbidden polarization angles $\theta_{\mathrm{f}}^{(L,R)}$ and the degree of enantiospecificity D as functions of the static E-field strength E_0, for 1,2-propanediol molecules with relevant quantum numbers $(\alpha = 1; \beta = 1; \gamma = 4)$ and $(\alpha = 1; \beta = 3; \gamma = 4)$. Here we illustrate $\theta_{\mathrm{f}}^{(L,R)}(E_0)$ and $D(E_0)$ for other two cases with $(\alpha = 1; \beta = 3; \gamma = 2)$ and $(\alpha = 1; \beta = 4; \gamma = 2)$ in Fig. S3.

S4 Analysis on the Origin of the Enantiospecific TPSR

In this section we show some details of the analysis on the origin of the enantiospecific TPSR.

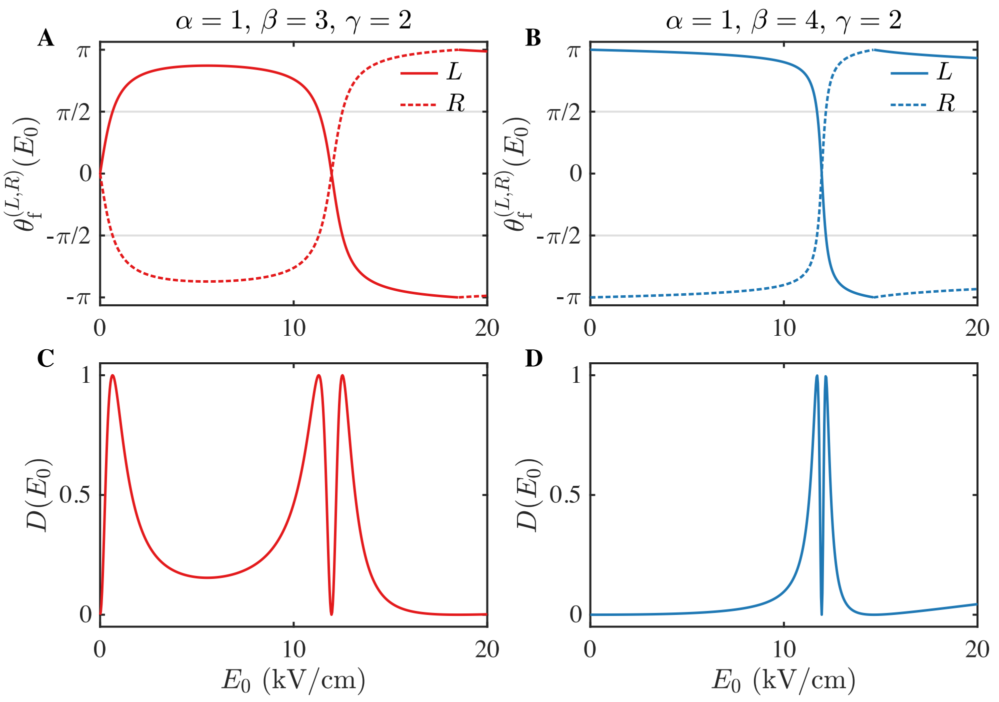

Figure S3: **Forbidden polarization angles** $\theta_{\rm f}^{(L,R)}$ **and enantiospecificity degree** D **of 1,2-propanediol for examples other than Fig. 2.** (**A** and **B**) The forbidden polarization angle $\theta_{\rm f}^{(L,R)}(E_0)$. (**C** and **D**) The degree $D(E_0)$ of enantiospecificity of the TPSR. Here we show the results for transitions of Eq. (1) for 1,2-propanediol molecules, with relevant quantum numbers being ($\alpha = 1; \beta = 3; \gamma = 2$) (**A** and **C**), and ($\alpha = 1; \beta = 4; \gamma = 2$) (**B** and **D**).

S4.1 Hamiltonian and Hilbert Space

As in the maintext, we denote x, y, z as the spatial coordinates of the particles (atomic nucleus and electrons) of each molecule, in the center-of-mass (CoM) frame of this molecule, e.g., $x := \{x_1, x_2, \ldots\}$, where x_i ($i = 1, 2, \ldots$) is the x-coordinate of the particle i. Note that the origin of the CoM frame is located at the CoM position of the molecule, and the axes of this frame are parallel to those of the lab frame. Each molecular state $|\xi, M; s\rangle$ is just a state of the relative motion of these particles, and corresponds to a wave function $\langle x, y, z|\xi, M; s\rangle$. We further denote $\mathscr{H}_s$ ($s = L, R$) as the Hilbert space spanned by $\{|\xi, M; s\rangle|_{E_0=0}\}$, i.e., the space of the states with chirality s.

In the total Hilbert space $\mathscr{H} = \mathscr{H}_L \oplus \mathscr{H}_R$, the Hamiltonian of the molecular internal state is

$$\hat{H}_0(E_0) = \hat{H}_{\rm F} - E_0 \hat{\boldsymbol{d}} \cdot \mathbf{e}_z, \tag{S13}$$

where $\hat{\boldsymbol{d}}$ is the molecular electric-dipole operator, and $\hat{H}_{\rm F}$ is the Hamiltonian in the absence of the static electric field, which includes the relative kinetic energy and interaction of the particles in the molecule. Additionally, $\hat{H}_{\rm F}$ is close in the subspaces $\mathscr{H}_L$ and $\mathscr{H}_R$, and can be expressed as $\hat{H}_{\rm F} = \hat{H}_{\rm F}^{(L)} + \hat{H}_{\rm F}^{(R)}$, where $\hat{H}_{\rm F}^{(s)}$ $(s = L, R)$ operates on the states in the subspace $\mathscr{H}_s$, and is studied in detail in Sec. S2.

Precisely speaking, the term $-E_0\hat{\boldsymbol{d}} \cdot \mathbf{e}_z$ in Eq. (S13) can induce not only the coupling between the states within the specific space $\mathscr{H}_{L(R)}$, but also the coupling between $\mathscr{H}_L$ and $\mathscr{H}_R$. However, as mentioned in our main text, we assume that E_0 is so weak that we can only consider the induced coupling between eigen-states of $\hat{H}_{\rm F}^{(L)}$ ($\hat{H}_{\rm F}^{(R)}$) within the same electronic and vibrational level. Clearly, under this approximation, the coupling between $\mathscr{H}_L$ and $\mathscr{H}_R$ is totally ignored, and we can express $\hat{H}_0(E_0)$ as $\hat{H}_0(E_0) = \hat{H}_0^{(L)}(E_0) + \hat{H}_0^{(R)}(E_0)$, as in the main text. Here $\hat{H}_0^{(s)}(E_0)$ $(s = L, R)$ operates on the states in the subspace $\mathscr{H}_s$. Accordingly, the eigen-state $|\xi,M;s\rangle$ of $\hat{H}_0^{(s)}(E_0)$, which is in the subspace $\mathscr{H}_s$, is also an eigen-state of $\hat{H}_0(E_0)$.

S4.2 Properties of the Transformations

For convenience of the following discussions, here we show some properties of the transformations $\hat{\mathcal{P}}$, $\hat{C}_{2x}$, and $\hat{\mathcal{T}}$ introduced in the main text.

(1) About the free Hamiltonian $\hat{H}_{\rm F}$:

$$\hat{C}_{2x}\hat{H}_{\rm F}\hat{C}_{2x} = \hat{\mathcal{P}}\hat{H}_{\rm F}\hat{\mathcal{P}} = \hat{\mathcal{T}}\hat{H}_{\rm F}\hat{\mathcal{T}} = \hat{H}_{\rm F}. \tag{S14}$$

(2) About the dipole operator:

$$\hat{\mathcal{P}}(\hat{\boldsymbol{d}} \cdot \mathbf{e}_{z,\pm})\hat{\mathcal{P}} = -\hat{\boldsymbol{d}} \cdot \mathbf{e}_{z,\pm}; \tag{S15}$$

$$\hat{C}_{2x}(\hat{\boldsymbol{d}} \cdot \mathbf{e}_z)\hat{C}_{2x} = -\hat{\boldsymbol{d}} \cdot \mathbf{e}_z; \tag{S16}$$

$$\hat{\mathcal{T}}(\hat{\boldsymbol{d}} \cdot \mathbf{e}_z)\hat{\mathcal{T}} = \hat{\boldsymbol{d}} \cdot \mathbf{e}_z; \tag{S17}$$

$$\hat{C}_{2x}(\hat{\boldsymbol{d}} \cdot \mathbf{e}_\pm)\hat{C}_{2x} = \hat{\mathcal{T}}(\hat{\boldsymbol{d}} \cdot \mathbf{e}_\pm)\hat{\mathcal{T}} = -\hat{\boldsymbol{d}} \cdot \mathbf{e}_\mp. \tag{S18}$$

(3) We define $|\mathcal{T}[\xi, M; s]\rangle$ as the result of applying the time-reversal operator $\hat{\mathcal{T}}$ to state $|\xi, M; s\rangle$, i.e.,

$$|\mathcal{T}[\xi, M; s]\rangle := \hat{\mathcal{T}}|\xi, M; s\rangle, \qquad (\xi = 1, 2, ...; M = 0, \pm 1; s = L, R). \tag{S19}$$

Thus,

$$\langle \boldsymbol{r}|\mathcal{T}[\xi, M; s]\rangle = \langle \boldsymbol{r}|\xi, M; s\rangle^*, \qquad (\xi = 1, 2, ...; M = 0, \pm 1; s = L, R). \tag{S20}$$

These results can be derived straightforwardly. Here we provide the following explanations of the derivation.

First, in deriving the results for time reversal operator $\hat{\mathcal{T}}$, i.e., in Eqs. (S17, S18, S20), we used the facts that for any operator $\hat{A}$, state $|\Psi\rangle$, and complex number λ, we have $\hat{\mathcal{T}}(\lambda\hat{A})\hat{\mathcal{T}} = \lambda^*\hat{\mathcal{T}}\hat{A}\hat{\mathcal{T}}$ and $\langle \mathbf{r}|\hat{\mathcal{T}}|\Psi\rangle = \langle \mathbf{r}|\Psi\rangle^*$. These facts arise because $\hat{\mathcal{T}}$ is an anti-linear operator in quantum mechanics, rather than a linear operator (*59*).

Second, the results in Eqs. (S16, S18), which are related to the rotation $\hat{C}_{2x}$ along the x-axis by π, can be directly understood through the physical interpretation of this rotation. Specifically, under this rotation, the direction vector $\mathbf{e}_x$ remains unchanged, while $\mathbf{e}_y$ and $\mathbf{e}_z$ are inverted. That is, we have $\hat{C}_{2x}\mathbf{e}_x\hat{C}_{2x} = \mathbf{e}_x, \hat{C}_{2x}\mathbf{e}_y\hat{C}_{2x} = -\mathbf{e}_y, \hat{C}_{2x}\mathbf{e}_z\hat{C}_{2x} = -\mathbf{e}_z$.

S4.3 Proof of the Relations $a_{\pm}^{(R)} = -a_{\pm}^{(L)*}$ and $b_{\pm}^{(R)} = -b_{\pm}^{(L)*}$

In this subsection we prove the relations $a_{\pm}^{(R)} = -a_{\pm}^{(L)*}$ and $b_{\pm}^{(R)} = -b_{\pm}^{(L)*}$, which are used to derive Eq. (7). As mentioned in the main text, the states $|\xi,M;R\rangle$ and $|\xi,M;L\rangle$ are related via $|\xi,M;R\rangle = \hat{\mathcal{P}}\hat{C}_{2x}\hat{\mathcal{T}}|\xi,M;L\rangle$. Substituting this result into Eq. (4) of the main text, we obtain

$$a_{+}^{(R)} = \langle \beta, +1; R|\hat{\boldsymbol{d}} \cdot \mathbf{e}_{+}|\alpha, 0; R\rangle = \int d\boldsymbol{r}\langle \mathcal{T}[\beta,+1;L]|\boldsymbol{r}\rangle \left[\hat{C}_{2x}\hat{\mathcal{P}}\left(\hat{\boldsymbol{d}} \cdot \mathbf{e}_{+}\right)\hat{\mathcal{P}}\hat{C}_{2x}\right]\langle \boldsymbol{r}|\mathcal{T}[\alpha,0;L]\rangle. \tag{S21}$$

On the other hand, Eqs. (S15, S18) yield

$$\hat{C}_{2x}\hat{\mathcal{P}}\left(\hat{\boldsymbol{d}} \cdot \mathbf{e}_{+}\right)\hat{\mathcal{P}}\hat{C}_{2x} = \hat{\boldsymbol{d}} \cdot \mathbf{e}_{-}. \tag{S22}$$

Substituting this result, Eq. (S20), and $\mathbf{e}_{-} = -\mathbf{e}_{+}^{*}$ into Eq. (S21), we obtain

$$a_{+}^{(R)} = \left[-\int d\boldsymbol{r}\langle \beta,+1;L|\boldsymbol{r}\rangle\left(\hat{\boldsymbol{d}} \cdot \mathbf{e}_{+}\right)\langle \boldsymbol{r}|\alpha,0;L\rangle\right]^* = -a_{+}^{(L)*}. \tag{S23}$$

Thus, we have proven $a_{+}^{(R)} = -a_{+}^{(L)*}$. The relations $a_{-}^{(R)} = -a_{-}^{(L)*}$ and $b_{\pm}^{(R)} = -b_{\pm}^{(L)*}$ can be proven with the same approach.

S4.4 Relation Between the Factor $a_+^{(s)*}b_-^{(s)*}b_+^{(s)}a_-^{(s)}$ and the Transformation $\hat{C}_{2x}\hat{\mathcal{T}}$

In this subsection we prove that the factor $a_+^{(s)*}b_-^{(s)*}b_+^{(s)}a_-^{(s)}$ is real when the Hamiltonian $\hat{H}_0^{(s)}(E_0)$ ($s = L, R$) is invariable under the combined transformation $\hat{C}_{2x}\hat{\mathcal{T}}$, i.e., when $[\hat{H}_0^{(s)}(E_0), \hat{C}_{2x}\hat{\mathcal{T}}] = 0$.

We notice that both the operators $\hat{H}_0^{(s)}(E_0)$ and $\hat{C}_{2x}\hat{\mathcal{T}}$ are close in the subspace with a fixed quantum number M of the total angular momentum $\hat{J}_z$, and in this subspace $\hat{H}_0^{(s)}(E_0)$ does not degenerate. Due to these facts, when $[\hat{H}_0^{(s)}(E_0), \hat{C}_{2x}\hat{\mathcal{T}}] = 0$, by choosing an appropriate global phase for the state $|\xi,M;s\rangle$, we can always make it an eigenstate of $\hat{C}_{2x}\hat{\mathcal{T}}$, with the eigenvalue being $+1$. That is,

$$\hat{C}_{2x}\hat{\mathcal{T}}|\xi,M;s\rangle = |\xi,M;s\rangle, \qquad (s = L, R). \tag{S24}$$

We choose the global phase of each state $|\xi,M;s\rangle$ ($\xi = 1, 2, \ldots;, M = 0, \pm1;, s = L, R$) such that Eq. (S24) is satisfied. Thus, according to Eqs. (S18, S20), we have

$$\begin{aligned} a_+^{(s)} &= \langle\beta, +1; s|\hat{\boldsymbol{d}} \cdot \mathbf{e}_+|\alpha, 0; s\rangle \\ &= \int d\boldsymbol{r}\langle\mathcal{T}[\beta,+1;s]|\boldsymbol{r}\rangle\left[\hat{C}_{2x}\left(\hat{\boldsymbol{d}} \cdot \mathbf{e}_+\right)\hat{C}_{2x}\right]\langle\boldsymbol{r}|\mathcal{T}[\alpha,0;s]\rangle \\ &= \left[\int d\boldsymbol{r}\langle\beta,+1;s|\boldsymbol{r}\rangle\left(\hat{\boldsymbol{d}} \cdot \mathbf{e}_+\right)\langle\boldsymbol{r}|\alpha,0;s\rangle\right]^* \\ &= a_+^{(s)*}. \end{aligned} \tag{S25}$$

Eq. (S25) yields that $a_+^{(s)}$ is *real*. With the same approach we can also prove that $a_-^{(s)}$ and $b_\pm^{(s)}$ are all real. Therefore, the factor $a_+^{(s)*}b_-^{(s)*}b_+^{(s)}a_-^{(s)}$ is real.

On the other hand, the value of $a_+^{(s)*}b_-^{(s)*}b_+^{(s)}a_-^{(s)}$ is independent of the choice of the global phases of the states $|\xi,M;s\rangle$ ($\xi = 1, 2, ...; M = 0, \pm1; s = L, R$). Therefore, although the above derivation is based on the global phases satisfying Eq. (S24), it actually leads to the conclusion that $a_+^{(s)*}b_-^{(s)*}b_+^{(s)}a_-^{(s)}$ is always real under the condition $[\hat{H}_0^{(s)}(E_0), \hat{C}_{2x}\hat{\mathcal{T}}] = 0$.

S4.5 Intuitive Picture of the Enantiospecific TPSR

In the end of this section, we provide an intuitive picture of the enantiospecific TPSR, which is simpler than the above explicit symmetry analysis. We first introduce the following abbreviation:

$\mathrm{T}[\boldsymbol{E}, \mathbf{e}_y, \mathbf{n}]$: The two-photon cascade transition of Eq. (1), with a static E-field $\boldsymbol{E}$, and beams 1 and 2 being polarized along $\mathbf{e}_y$ and $\mathbf{n}$, respectively.

In addition, we define the vector-valued function $\mathbf{e}_2(\theta)$ as follows:

$$\mathbf{e}_2(\theta) = \sin\left(\frac{\theta}{2}\right)\mathbf{e}_x - \cos\left(\frac{\theta}{2}\right)\mathbf{e}_y.$$

We begin our analysis from a single left-handed molecule, i.e., molecule L. As mentioned in our main text, we defined the forbidden polarization angle $\theta_{\mathrm{f}}^{(L)}(E_0)$ $(E_0 > 0)$ such that (Fig. S4):

$$\mathrm{T}\left[E_0\mathbf{e}_z, \mathbf{e}_y, \mathbf{e}_2\left(\theta_{\mathrm{f}}^{(L)}(E_0)\right)\right] \text{ is forbidden for molecule } L. \qquad \text{(Fact 1)}$$

Furthermore, we consider the two-photon cascade transition under a reversed static electric field, $-E_0\mathbf{e}_z$. In this case, we define a new set of axes for the laboratory frame: $\mathbf{e}'_x = -\mathbf{e}_x, \mathbf{e}'_y = \mathbf{e}_y, \mathbf{e}'_z = -\mathbf{e}_z$. Note that the triplet $(\mathbf{e}'_x, \mathbf{e}'_y, \mathbf{e}'_z)$ still forms a right-handed coordinate system, and the new electric field $-E_0\mathbf{e}_z$ now points in the positive direction of $\mathbf{e}'_z$. Thus, for arbitrary direction $\mathbf{n}$, the transition $\mathrm{T}[-E_0\mathbf{e}_z, \mathbf{e}_y, \mathbf{n}]$ can be re-expressed as $\mathrm{T}[E_0\mathbf{e}'_z, \mathbf{e}'_y, \mathbf{n}]$. According to the definition of $\theta_{\mathrm{f}}^{(L)}$ shown above, this transition is forbidden for molecule L when

$$\mathbf{n} = \sin\left(\frac{\theta_{\mathrm{f}}^{(L)}(E_0)}{2}\right)\mathbf{e}'_x - \cos\left(\frac{\theta_{\mathrm{f}}^{(L)}(E_0)}{2}\right)\mathbf{e}'_y = \mathbf{e}_2\left(-\theta_{\mathrm{f}}^{(L)}(E_0)\right).$$

In other words, we have (Fig. S4):

$$\mathrm{T}\left[-E_0\mathbf{e}_z, \mathbf{e}_y, \mathbf{e}_2\left(-\theta_{\mathrm{f}}^{(L)}(E_0)\right)\right] \text{ is forbidden for molecule } L. \qquad \text{(Fact 2)}$$

Moreover, the above two facts (Fact 1 and Fact 2) imply that, in the absence of the static electric field ($E_0 = 0$), the two-photon transition is forbidden when beam 2 is polarized along either $\mathbf{e}_2\left(\theta_{\mathrm{f}}^{(L)}(0)\right)$ or $\mathbf{e}_2\left(-\theta_{\mathrm{f}}^{(L)}(0)\right)$. On the other hand, in our system—which involves two intermediate β-states—there exists only one specific polarization of beam 2 that renders the transition forbidden. Therefore, the two directions must be equivalent, i.e., $\mathbf{e}_2\left(\theta_{\mathrm{f}}^{(L)}(0)\right) = \pm\mathbf{e}_2\left(-\theta_{\mathrm{f}}^{(L)}(0)\right)$, which leads to the conclusion that $\theta_{\mathrm{f}}^{(L)}(0)$ must be either 0 or π. Furthermore, in the presence of a static electric field ($E_0 \neq 0$), the forbidden polarization angle $\theta_{\mathrm{f}}^{(L)}(E_0)$ generally takes values different from 0 or π.

Now let us consider the right-handed molecule R. Intuitively speaking, if a physical process is forbidden for molecule L, then the spatially inverted version of that process is also forbidden for

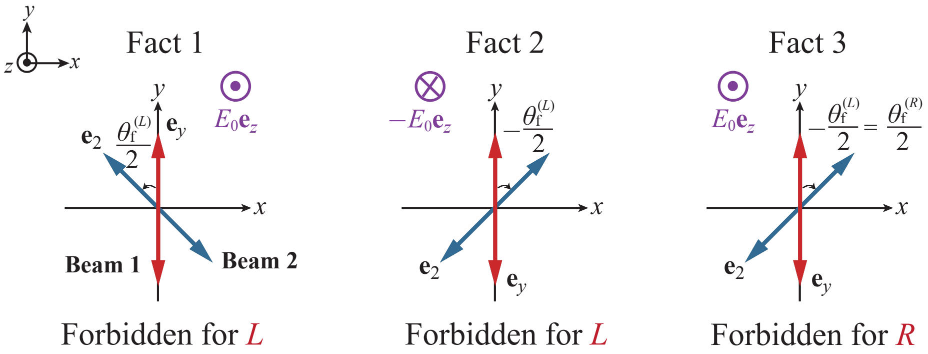

Figure S4: **The Facts 1, 2, and 3 mentioned in Sec. S4.5.**

molecule R. Therefore, according to the above (Fact 2), the transition $\mathrm{T}\left[E_0\mathbf{e}_z, -\mathbf{e}_y, -\mathbf{e}_2\left(-\theta_{\mathrm{f}}^{(L)}(E_0)\right)\right]$ ($E_0 \geq 0$) is forbidden for molecule R. Furthermore, note that for a beam, two polarization directions that differ by a sign are equivalent. Thus, we conclude (Fig. S4):

$$\mathrm{T}\left[E_0\mathbf{e}_z, \mathbf{e}_y, \mathbf{e}_2\left(-\theta_{\mathrm{f}}^{(L)}(E_0)\right)\right] \text{ is forbidden for molecule } R. \qquad \text{(Fact 3)}$$

On the other hand, the forbidden polarization angle $\theta_{\mathrm{f}}^{(R)}(E_0)$ is defined to satisfy:

$$\mathrm{T}\left[E_0\mathbf{e}_z, \mathbf{e}_y, \mathbf{e}_2\left(\theta_{\mathrm{f}}^{(R)}(E_0)\right)\right] \text{ is forbidden for molecule } R. \qquad \text{(Fact 4)}$$

Combining (Fact 3) and (Fact 4), we arrive at the relation: $\theta_{\mathrm{f}}^{(R)}(E_0) = -\theta_{\mathrm{f}}^{(L)}(E_0)$. Moreover, as discussed earlier, when $E_0 \neq 0$, the forbidden angle $\theta_{\mathrm{f}}^{(L)}(E_0)$ generally takes values other than 0 or π. Combining this observation with the result above, we find that for $E_0 \neq 0$, we generally have $\mathbf{e}_2\left(\theta_{\mathrm{f}}^{(R)}(E_0)\right) \neq \pm\mathbf{e}_2\left(-\theta_{\mathrm{f}}^{(L)}(E_0)\right)$. In other words, the polarization of beam 2, for which the two-photon transition is forbidden, is different for the molecules L and R. That is the intuitive picture of the enantiospecific TPSR.

S5 Calculation for the Results of Fig. 3

Now we show our approach for the calculation of the cascade-transition dynamics, i.e., the results of Fig. 3.

S5.1 The Final-State Probability $P_\gamma^{(s)}$

As mentioned in the main text, we consider the case with $\epsilon_{\alpha,0} < \epsilon_{\beta,\pm1} < \epsilon_{\gamma,0}$. Moreover, the electric field strength $\boldsymbol{\mathcal{E}}_{1(2)}$ of beams 1 and 2 can be expressed as

$$\boldsymbol{\mathcal{E}}_1 = \mathcal{E}_1 e^{-i\omega_1 t}\mathbf{e}_y + c.c.; \quad \boldsymbol{\mathcal{E}}_2 = \mathcal{E}_2 e^{-i\omega_2 t}\mathbf{e}_2 + c.c., \tag{S26}$$

where $\mathcal{E}_{1,2}$ are corresponding complex amplitudes. When these beams are applied, in the rotating frame the Hamiltonian of the molecule s ($s = L, R$) is given by ($\hbar = 1$):

$$\hat{H}^{(s)} = \Delta_1 \sum_{M=\pm1} |\beta,M;s\rangle\langle\beta,M;s| + (\Delta_1 + \Delta_2)|\gamma,0;s\rangle\langle\gamma,0;s| + \hat{H}_{\mathrm{I}1} + \hat{H}_{\mathrm{I}2}, \tag{S27}$$

where $\Delta_1 = \epsilon_{\beta,1} - \epsilon_{\alpha,0} - \omega_1$ and $\Delta_1 + \Delta_2 = \epsilon_{\gamma,0} - \epsilon_{\alpha,0} - (\omega_1 + \omega_2)$ are the one-photon and two-photon detunings, respectively, with $\omega_{1(2)}$ being the angular frequency of the beam 1 (2). Additionally, $\hat{H}_{\mathrm{I}1(2)}$ is the interaction between the beam 1 (2) and the molecule, and can be expressed as

$$\begin{aligned}\hat{H}_{\mathrm{I}1} = &-\frac{\mathcal{E}_1}{\sqrt{2}}\left\{a_+^{(s)}|\beta,+1;s\rangle\langle\alpha,0;s| + a_-^{(s)}|\beta,-1;s\rangle\langle\alpha,0;s|\right\} \\ &-\frac{\mathcal{E}_1^*}{\sqrt{2}}\left\{a_+^{(s)*}|\alpha,0;s\rangle\langle\beta,+1;s| + a_-^{(s)*}|\alpha,0;s\rangle\langle\beta,-1;s|\right\},\end{aligned} \tag{S28}$$

and

$$\begin{aligned}\hat{H}_{\mathrm{I}2} = &-\frac{\mathcal{E}_2}{\sqrt{2}}\left\{b_-^{(s)}e^{i\theta}|\gamma,0;s\rangle\langle\beta,+1;s| + b_+^{(s)}|\gamma,0;s\rangle\langle\beta,-1;s|\right\} \\ &-\frac{\mathcal{E}_2^*}{\sqrt{2}}\left\{b_-^{(s)*}e^{-i\theta}|\beta,+1;s\rangle\langle\gamma,0;s| + b_+^{(s)*}|\beta,-1;s\rangle\langle\gamma,0;s|\right\}.\end{aligned} \tag{S29}$$

Note that $\hat{H}_{\mathrm{I}2}$ depends on the angle θ, due to the fact $\mathbf{e}_2 \propto (\mathbf{e}_+ + e^{i\theta}\mathbf{e}_-)$.

For the convenience of the following calculations, we define the Rabi frequencies (coupling strengths):

$$\Omega_1 = \sqrt{2}\left|\mathcal{E}_1 a_+^{(s)}\right| = \sqrt{2}\left|\mathcal{E}_1 a_-^{(s)}\right|; \qquad \Omega_2 = \sqrt{2}\left|\mathcal{E}_2 b_+^{(s)}\right| = \sqrt{2}\left|\mathcal{E}_2 b_-^{(s)}\right|. \tag{S30}$$

Here we have used the facts $|a_+^{(L)}| = |a_-^{(L)}| = |a_+^{(R)}| = |a_-^{(R)}|$, and $|b_+^{(L)}| = |b_-^{(L)}| = |b_+^{(R)}| = |b_-^{(R)}|$, which are due to the the time-reversal symmetry and the relations $a_\pm^{(R)} = -a_\pm^{(L)^*}$ and $b_\pm^{(R)} = -b_\pm^{(L)^*}$ proven in Sec. S4.3. Note that the Rabi frequencies Ω_1 and Ω_2 are independent of the molecular chirality. We further define the phases

$$\phi_{1\pm}^{(s)} = \arg\left[-\mathcal{E}_1 a_\pm^{(s)}\right]; \qquad \phi_{2\pm}^{(s)} = \arg\left[-\mathcal{E}_2 b_\pm^{(s)}\right]. \tag{S31}$$

Using Eq. (7) of the main text, we find that these angles are related to the forbidden polarization angle $\theta_{\mathrm{f}}^{(s)}$ via

$$e^{i\left(\phi_{2+}^{(s)}+\phi_{1-}^{(s)}-\phi_{1+}^{(s)}-\phi_{2-}^{(s)}\right)} = -e^{i\theta_{\mathrm{f}}^{(s)}(E_0)}. \tag{S32}$$

We further introduce the states

$$\overline{|\beta, \pm 1; s\rangle} = |\beta, \pm 1; s\rangle e^{i\phi_{1\pm}^{(s)}}; \tag{S33}$$

$$\overline{|\gamma, 0; s\rangle} = |\gamma, 0; s\rangle e^{i\left(\phi_{1+}^{(s)}+\phi_{2-}^{(s)}\right)}. \tag{S34}$$

In the basis $\{|\alpha, 0; s\rangle, \overline{|\beta, \pm 1; s\rangle}, \overline{|\gamma, 0; s\rangle}\}$, the total Hamiltonian $\hat{H}^{(s)}$ of Eq. (S27) can be re-written as

$$\begin{aligned}\hat{H}^{(s)} &= \Delta_1 \sum_{M=\pm 1} \overline{|\beta,M;s\rangle}\overline{\langle\beta,M;s|} + (\Delta_1+\Delta_2)\overline{|\gamma,0;s\rangle}\overline{\langle\gamma,0;s|} \\ &+\left(\frac{\Omega_1}{2}\left[\overline{|\beta,+1;s\rangle}\langle\alpha,0;s| + \overline{|\beta,-1;s\rangle}\langle\alpha,0;s|\right] + \frac{\Omega_2}{2}\left[e^{i\theta}\overline{|\gamma,0;s\rangle}\overline{\langle\beta,+1;s|} - e^{i\theta_{\mathrm{f}}^{(s)}(E_0)}\overline{|\gamma,0;s\rangle}\overline{\langle\beta,-1;s|}\right]\right. \\ &\left.+ h.c.\right), \qquad (s = L, R).\end{aligned} \tag{S35}$$

Here we have used Eq. (S32).

In our calculation, we express the time-dependent state $|\Psi^{(s)}(t)\rangle$ of molecule s ($s = L, R$) as

$$|\Psi^{(s)}(t)\rangle = c_{\alpha}^{(s)}(t)|\alpha, 0; s\rangle + c_{\beta+}^{(s)}(t)\overline{|\beta, +1; s\rangle} + c_{\beta-}^{(s)}(t)\overline{|\beta, -1; s\rangle} + c_{\gamma}^{(s)}(t)\overline{|\gamma, 0; s\rangle}. \tag{S36}$$

Substituting this expression and Eq. (S35) into the Schrödinger equation

$$i\frac{d}{dt}|\Psi^{(s)}(t)\rangle = \hat{H}^{(s)}|\Psi^{(s)}(t)\rangle, \tag{S37}$$

we can obtain the equations for the coefficients $c_{\alpha,\beta\pm,\gamma}^{(s)}(t)$ as

$$\dot{c}_{\alpha}^{(s)} = -i\frac{\Omega_1}{2}c_{\beta+}^{(s)} - i\frac{\Omega_1}{2}c_{\beta-}^{(s)}, \tag{S38}$$

$$\dot{c}_{\beta+}^{(s)} = -i\frac{\Omega_1}{2}c_{\alpha}^{(s)} - i\Delta_1 c_{\beta+}^{(s)} - i\frac{\Omega_2}{2}e^{-i\theta}c_{\gamma}^{(s)}, \tag{S39}$$

$$\dot{c}_{\beta-}^{(s)} = -i\frac{\Omega_1}{2}c_{\alpha}^{(s)} - i\Delta_1 c_{\beta-}^{(s)} + i\frac{\Omega_2}{2}e^{-i\theta_{\mathrm{f}}^{(s)}}c_{\gamma}^{(s)}, \tag{S40}$$

$$\dot{c}_{\gamma}^{(s)} = -i\frac{\Omega_2}{2}e^{i\theta}c_{\beta+}^{(s)} + i\frac{\Omega_2}{2}e^{i\theta_{\mathrm{f}}^{(s)}}c_{\beta-}^{(s)} - i(\Delta_1+\Delta_2)c_{\gamma}^{(s)}. \tag{S41}$$

Solving this equation with initial condition $c_{\alpha}^{(s)}(0) = 1$ and $c_{\beta\pm,\gamma}^{(s)}(0) = 0$ (corresponding to the initial state $|\Psi^{(s)}(0)\rangle = |\alpha, 0; s\rangle$), we can obtain the time evolution of the coefficient $c_{\gamma}^{(s)}(t)$ for the final state $|\gamma, 0; s\rangle$, and the final-state probability $P_{\gamma}^{(s)}(t)$ which is given by $P_{\gamma}^{(s)}(t) = |c_{\gamma}^{(s)}(t)|^2$.

Finally, we address the following question: When the $\alpha \to \gamma$ transition is forbidden for one enantiomer, how can we ensure that a sufficiently large portion of the other enantiomer is excited to the final state? We consider the case where $\theta = \theta_{\mathrm{f}}^{(L)}$ (i.e., the $\alpha \to \gamma$ transition is forbidden for molecule L) as an example. From our previous discussion, it follows that in this case, the final-state probability $P_{\gamma}^{(R)}(t)$ of molecule R is still given by $P_{\gamma}^{(R)}(t) = |c_{\gamma}^{(R)}(t)|^2$, where $c_{\gamma}^{(R)}(t)$ is the solution of Eqs. (S38-S41), with $s = R$ and $\theta = \theta_{\mathrm{f}}^{(L)}$. Since these equations are independent of the phases of beams 1 and 2, phase-locking is not required to control $P_{\gamma}^{(R)}(t)$. Specifically, according to these equations, $P_{\gamma}^{(R)}(t)$ can be tuned by adjusting the Rabi frequencies $\Omega_{1,2}$ and the detunings $\Delta_{1,2}$, which are controlled through the intensities and frequencies of the beams 1 and 2, respectively. The conditions for $\Omega_{1,2}$ and $\Delta_{1,2}$ required to optimize $P_{\gamma}^{(R)}(t)$ can be derived by solving these equations.

5.1.1. The Essential Role of the Degeneracy of $|\beta, \pm 1; s\rangle$

The degeneracy of the β-states $|\beta, \pm 1; s\rangle$ is essential for the emergence of the TPSR. We can understand this with calculations in the above framework. Specifically, we consider the case where $\theta = \theta_{\mathrm{f}}^{(L)}$, and assume that the energies of the states $|\beta, \pm 1; s\rangle$ are non-degenerate, i.e., $\epsilon_{\beta,+1} \neq \epsilon_{\beta,-1}$. Under this assumption, in the basis $\{|\alpha, 0; L\rangle, |\overline{\beta, \pm 1; L}\rangle, |\overline{\gamma, 0; L}\rangle\}$, the expression for the total Hamiltonian $\hat{H}^{(L)}$ of molecule L, originally expressed in Eq. (S27), must be modified to

$$
\begin{aligned}
&\hat{H}^{(L)} \\
= \;& \Delta_1 |\overline{\beta, +1; L}\rangle\langle\overline{\beta, +1; L}| + (\Delta_1 + \Delta_2)|\overline{\gamma, 0; L}\rangle\langle\overline{\gamma, 0; L}| + (\Delta_1 + \delta_\beta)|\overline{\beta, -1; L}\rangle\langle\overline{\beta, -1; L}| \\
& + \Bigg(\frac{\Omega_1}{2} \left[|\overline{\beta, +1; L}\rangle\langle\alpha, 0; L| + |\overline{\beta, -1; L}\rangle\langle\alpha, 0; L| \right] + \frac{\Omega_2}{2} \Big[e^{i\theta_{\mathrm{f}}^{(L)}} |\overline{\gamma, 0; L}\rangle\langle\overline{\beta, +1; L}| \\
& - e^{i\theta_{\mathrm{f}}^{(L)}} |\overline{\gamma, 0; L}\rangle\langle\overline{\beta, -1; L}| \Big] + h.c. \Bigg),
\end{aligned}
\tag{S42}
$$

where $\Delta_1 = \epsilon_{\beta,+1} - \epsilon_{\alpha,0} - \omega_1$ and $\Delta_1 + \Delta_2 = \epsilon_{\gamma,0} - \epsilon_{\alpha,0} - (\omega_1 + \omega_2)$, as defined before, and $\delta_\beta = \epsilon_{\beta,+1} - \epsilon_{\beta,-1}$. We further introduce states

$$
|\overline{\psi_\pm; L}\rangle = \frac{1}{\sqrt{2}} \left[|\overline{\beta, +1; L}\rangle \pm |\overline{\beta, -1; L}\rangle \right], \tag{S43}
$$

which form a group of new basis in the subspace spanned by $\{|\overline{\beta, \pm 1; L}\rangle\}$. In the basis $\{|\alpha, 0; L\rangle, |\overline{\psi_\pm; L}\rangle, |\overline{\gamma, 0; L}\rangle\}$, the Hamiltonian $\hat{H}^{(L)}$ can be re-expressed as

$$\begin{aligned}
\hat{H}^{(L)} &= \left(\Delta_1 + \frac{\delta_\beta}{2}\right)\left(|\overline{\psi_+; L}\rangle\langle\overline{\psi_+; L}| + |\overline{\psi_-; L}\rangle\langle\overline{\psi_-; L}|\right) + (\Delta_1 + \Delta_2)|\overline{\gamma, 0; L}\rangle\langle\overline{\gamma, 0; L}| \\
&\quad + \frac{\Omega_1}{\sqrt{2}}\left(|\overline{\psi_+; L}\rangle\langle\alpha, 0; L| + |\alpha, 0; L\rangle\langle\overline{\psi_+; L}|\right) \\
&\quad + \frac{\Omega_2}{\sqrt{2}}\left(e^{i\theta_{\mathrm{f}}^{(L)}}|\overline{\gamma, 0; L}\rangle\langle\overline{\psi_-; L}| + e^{-i\theta_{\mathrm{f}}^{(L)}}|\overline{\psi_-; L}\rangle\langle\overline{\gamma, 0; L}|\right) \\
&\quad - \frac{\delta_\beta}{2}\left(|\overline{\psi_+; L}\rangle\langle\overline{\psi_-; L}| + \mathrm{h.c.}\right). \qquad \text{(S44)}
\end{aligned}$$

Thus, when the states $|\overline{\beta, \pm 1; L}\rangle$ are degenerate (i.e., $\delta_\beta = 0$), the intermediate states $|\overline{\psi_\pm; L}\rangle$ are not coupled to each other, and the transition from the α-state to the γ-state is completely forbidden. However, if the degeneracy is lifted (i.e., $\delta_\beta \neq 0$), the states $|\overline{\psi_\pm; L}\rangle$ become effectively coupled via δ_β, as indicated by the last term in Eq. (S44). This coupling enables the system to undergo a transition from the α-state to the γ-state through a two-photon cascade. In this case, the transition is allowed, and the TPSR does not occur.

In conclusion, our analysis demonstrates that the presence of degeneracy in the states $|\overline{\beta, \pm 1; L}\rangle$ is essential for the appearance of TPSR in our system.

S5.2 Absorption A of Beam 2

Now we show our calculation for the results in Fig. 3D, i.e., the absorption $A^{(L,R)}(\theta)$ of beam 2, of left- and right-handed enantiomers. As mentioned in our maintext, we consider the system of Sec. S5.1, with beam 1 and beam 2 being a strong driving field and a weak probe field, respectively. We consider a case where $\epsilon_{\alpha,0} < \epsilon_{\beta,\pm 1} < \epsilon_{\gamma,0}$, and the spontaneous emissions from both the γ-state and the β-state are all non-negligible. For simplicity, in our model we take the rates of these spontaneous emissions are all κ. The Lindblad master equation of the molecule s ($s = L, R$) is

$$\begin{aligned}
\frac{d\hat{\rho}^{(s)}(t)}{dt} &= -i[\hat{H}^{(s)}, \hat{\rho}^{(s)}(t)] + \kappa\mathcal{L}[|\overline{\beta, +1; s}\rangle\langle\overline{\gamma, 0; s}|]\hat{\rho}^{(s)}(t) + \kappa\mathcal{L}[|\overline{\beta, -1; s}\rangle\langle\overline{\gamma, 0; s}|]\hat{\rho}^{(s)}(t) \\
&\quad + \kappa\mathcal{L}[|\alpha, 0; s\rangle\langle\overline{\gamma, 0; s}|]\hat{\rho}^{(s)}(t) + \kappa\mathcal{L}[|\alpha, 0; s\rangle\langle\overline{\beta, +1; s}|]\hat{\rho}^{(s)}(t) \\
&\quad + \kappa\mathcal{L}[|\alpha, 0; s\rangle\langle\overline{\beta, -1; s}|]\hat{\rho}^{(s)}(t), \qquad \text{(S45)}
\end{aligned}$$

where $\hat{H}^{(s)}$ is given in Eq. (S35), and the Lindblad superoperators used in Eq. (S45) are defined by

$$\mathcal{L}[|o_1\rangle\langle o_2|]\hat{\rho}^{(s)}(t) = |o_1\rangle\langle o_2|\hat{\rho}^{(s)}(t)|o_2\rangle\langle o_1| - \frac{1}{2}|o_2\rangle\langle o_2|\hat{\rho}^{(s)}(t) - \frac{1}{2}\hat{\rho}^{(s)}(t)|o_2\rangle\langle o_2|. \tag{S46}$$

By numerically solving Eq. (S45), we can obtain the steady-state density operator $\hat{\rho}_{\rm ss}^{(s)}$ of the molecule s, and the absorption of the beam 2 which is given by (*60*)

$$A^{(s)} = \lim_{\Omega_2\to 0} \frac{2n^{(s)}d_0^2\omega_2}{c\varepsilon_0}{\rm Im}\left[\frac{{\rm Tr}[\hat{\rho}_{\rm ss}^{(s)}\hat{d}_+^{(s)}]}{\Omega_2}\right], \qquad (s = L, R) \tag{S47}$$

with $\hat{d}_+^{(s)} = e^{-i\theta_{\rm f}^{(s)}}|\overline{\beta, -1; s}\rangle\langle\overline{\gamma, 0; s}| - e^{-i\theta}|\overline{\beta, +1; s}\rangle\langle\overline{\gamma, 0; s}|$, d_0 being the electric dipole moment of the $\beta \to \gamma$ transition, $n^{(s)}$ being the number density of the molecules s, ε_0 being the vacuum permittivity, ω_2 and c being the angular frequency of the beam 2 and the light speed in vacuum, respectively.

S6 Comparison of the Results of Fig. 3(A and B) and the Ones Obtained via Three-Microwave Methods

Figure 3(A and B) of our main text demonstrates the time evolution for the case with enantiospecific transition. It is shown that the final-state (γ-state) probability (FSP) of the enantiomer with a forbidden transition (denoted as s) remains zero at all times, while the FSP of the enantiomer with the opposite chirality (denoted as $\bar{s}$) is finite. Thus, for any mixture of enantiomers, in this evolution, all the molecules in the γ-state definitely have chirality $\bar{s}$. In other words, during the enantiospecific transitions, the state-specific enantiomeric purity of the final state (γ-state) remains 100% at all times, except at a few special time points with $P_\gamma^{(L)} = P_\gamma^{(R)} = 0$.

On the other hand, as discussed in the main text, coherent enantiospecific evolutions have also been proposed [Refs. (21–35) in the main text] and experimentally demonstrated [Refs. (37, 40, 43) of the main text] using three phase-locked microwave beams. In these evolutions, the state-specific enantiomeric purity of a given rotational state can, in theory, reach 100% at certain time instants. In state-of-the-art experiments, a state-specific enantiomeric purity of up to 96% has been achieved for a racemic mixture [Ref. (43) of the main text].

However, as mentioned above, in the three-microwave proposals the 100% state-specific enantiomeric purity appears only at certain time instants, whereas in our case it remains zero at all

times. As a result, those schemes demand precise control of the beam–molecule interaction time, beam frequencies, beam intensities, and the phase locking of the three beams—all of which are not required in our present approach.

In addition, we have recently proposed an enantiomer-pumping scheme [Ref. (36) in the main text] based on an incoherent dissipative process. In that scheme, the state-specific enantiomeric purity can reach 100% in the long-time limit, rather than being zero only at specific time instants. Thus, precise control of the beam–molecule interaction time is no longer necessary. Nevertheless, phase locking and accurate control of the beam frequencies and intensities are still required.

S7 Experimental Feasibility of Realization of TPSR

S7.1 Realization of the Static E-Field

As demonstrated in the main text and in Sec. S3, TPSR with significant enantiospecificity ($D \sim 1$) can occur with a static electric field strength $E_0 \leq 20\,\mathrm{kV/cm}$ for chiral molecules with rotational parameters A, B, and C [defined in Eq. (S5)] on the order of $\hbar(2\pi)$ GHz, and an electric dipole moment on the order of Debye. Additionally, for molecules with lower rotational parameters or higher electric dipole moments, the required value of E_0 to achieve $D \sim 1$ can be further reduced.

The static electric field with $E_0 \leq 20\,\mathrm{kV/cm}$ can be generated, for example, using a pair of parallel plate electrodes with a voltage of at most around 20 kV and a separation distance of approximately 1 cm, such as the setup used in Ref. (*61*) (voltage 18 kV, inter-plate distance 0.8 cm).

Such a static E-field is significantly lower than both the molecular ionization threshold ($\sim 10^5$ kV/cm) and the air breakdown field (~30 kV/cm), making ionization or air breakdown unlikely. However, whether breakdown occurs in a specific molecular sample depends on its properties and must be assessed experimentally. Importantly, as long as the static E-field is nonzero (except at specific values), TPSR retains enantiospecificity. Therefore, even if a weaker E-field must be used in experiments to prevent the sample from breaking down, it will at most reduce the enantiospecificity of TPSR, but it will not completely eliminate it, allowing enantiospecific transitions to still be realized.

S7.2 Sensitivity of $\theta_{\mathrm{f}}^{(L,R)}$ to Errors in Control Parameters

The forbidden polarization angles $\theta_{\mathrm{f}}^{(L,R)}$ of beam 2 are determined by the static E-field and the polarization of beam 1. We now examine the impact of errors in these parameters that arise in realistic experiments.

In the main text and previous sections of the Supplementary Materials, we assume that the static electric field is given by $\boldsymbol{E} = E_0\mathbf{e}_z$. However, in realistic scenarios, errors can occur in both the magnitude and direction of this field, arising from factors such as field inhomogeneity or misalignment. Thus, we have $\boldsymbol{E} = E_0\mathbf{e}_z + \delta\boldsymbol{E}$, where $\delta\boldsymbol{E}$ represents the error in $\boldsymbol{E}$, with the components (x, y, z) denoted as $\delta E_{x,y,z}$.

Additionally, we have assumed that beam 1 is polarized along the y-direction. In practical experiments, however, there may be errors in this polarization. Specifically, beam 1 is actually polarized along

$$\mathbf{e}_{1\mathrm{real}} := \cos\delta\phi\left[\cos(\delta\varkappa/2)\mathbf{e}_y + \sin(\delta\varkappa/2)\mathbf{e}_x\right] + \sin\delta\phi\mathbf{e}_z. \tag{S48}$$

Note that when $\delta\varkappa = \delta\phi = 0$, we have $\mathbf{e}_{1\mathrm{real}} = \mathbf{e}_y$. Thus, $\delta\varkappa$ and $\delta\phi$ represent the errors in polarization within the x-y plane and along the z-direction, respectively.

Both the electric field errors $\delta E_{x,y,z}$ and the beam-1 polarization errors $(\delta\varkappa, \delta\phi)$ can modify $\theta_{\mathrm{f}}^{(s)}$ $(s = L, R)$ from its designed value $\theta_{\mathrm{f}}^{(s)}(E_0)$. Specifically, we have:

$$\theta_{\mathrm{f}}^{(s)} = \theta_{\mathrm{f}}^{(s)}(E_0) + \delta\theta_{\mathrm{f}}^{(s)}, \tag{S49}$$

where $\delta\theta_{\mathrm{f}}^{(s)}$ represents the error in $\theta_{\mathrm{f}}^{(s)}$. Furthermore, when $\delta E_{x,y,z}$ and $(\delta\varkappa, \delta\phi)$ are sufficiently small, $\delta\theta_{\mathrm{f}}^{(s)}$ can be approximated as a linear function of these errors, i.e.,

$$\delta\theta_{\mathrm{f}}^{(s)} \approx \delta E_z \mathrm{S}_z^{(s)} + \delta E_x \mathrm{S}_x^{(s)} + \delta E_y \mathrm{S}_y^{(s)} + \delta\varkappa \mathrm{S}_\varkappa^{(s)} + \delta\phi \mathrm{S}_\phi^{(s)}, \quad (s = L, R). \tag{S50}$$

The sensitivity of $\theta_{\mathrm{f}}^{(s)}$ $(s = L, R)$ to these errors can be described by the coefficients $\mathrm{S}_{x,y,z,\varkappa,\phi}^{(s)}$.

Now we investigate the magnitudes of $\mathrm{S}_{x,y,z,\varkappa,\phi}^{(s)}$ $(s = L, R)$. Notice that the coupling between the E-field error $\delta\boldsymbol{E}$ and the molecule is described by $-\delta\boldsymbol{E}\cdot\hat{\boldsymbol{d}}$, with $\hat{\boldsymbol{d}}$ being the electric dipole moment operator of the molecule, as defined before. Similarly, the coupling between the error in beam 1 and the molecule is described by $-(\mathcal{E}_1 e^{-i\omega_1 t}\delta\mathbf{e} + c.c.)\cdot\hat{\boldsymbol{d}}$, where $\delta\mathbf{e} := \mathbf{e}_{1\mathrm{real}} - \mathbf{e}_y$. By incorporating

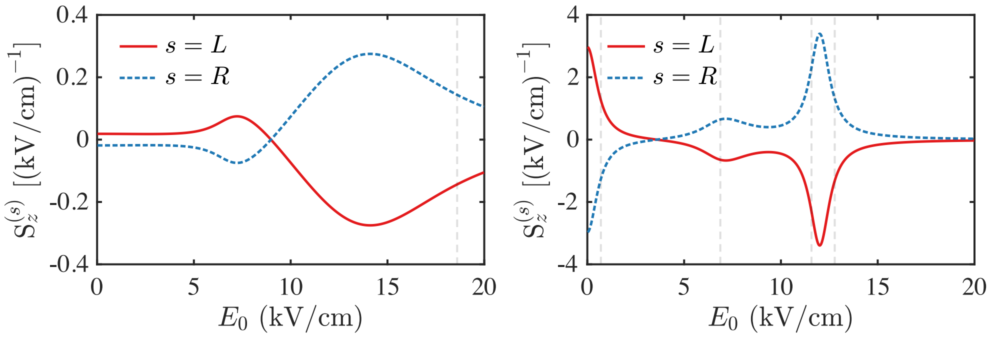

Figure S5: **The coefficient $S_z^{(L,R)}$ for the systems in Fig. 2A (left) and Fig. 2B (right).** The vertical dashed lines represent the values of E_0 at which the enantiospecificity of TPSR is maximized ($D = 1$).

these two coupling Hamiltonians into our calculation, we can derive $\theta_f^{(s)}$ ($s = L, R$) up to the first order of $\delta E_{x,y,z}$ and $(\delta\varkappa, \delta\phi)$, from which we obtain $S_{x,y,z,\varkappa,\phi}^{(s)}$. This approach directly yields

$$S_x^{(s)} = S_y^{(s)} = S_\phi^{(s)} = 0; \quad S_\varkappa^{(s)} = 1; \qquad (s = L, R). \tag{S51}$$

Thus, $\theta_f^{(s)}$ is robust with respect to the E-field error in the x-y plane, as well as the polarization error of beam 1 in the z-direction. Additionally, the induced error for $\theta_f^{(s)}$ will be much less than one radian, provided that the amplitude of the error $\delta\phi$ of the polarization of beam 1 in the x-y plane is much less than one radian. This condition is achievable in current experiments.

Finally, let us consider the coefficient $S_z^{(s)}$, which can be rewritten as $S_z^{(s)} = \frac{d\theta_f^{(s)}(E_0)}{dE_0}$. As a typical example, in Fig. S5 we present $S_z^{(s)}$ for the systems shown in Fig. 2. It is demonstrated that $S_z^{(s)}$ is on the order of 1 (kV/cm)$^{-1}$ to 0.1 (kV/cm)$^{-1}$. Thus, when δE_z is much less than 1 kV/cm to 10 kV/cm, the induced error in $\theta_f^{(s)}$ is much less than one radian.

This result establishes the requirement for the homogeneity of the static E-field in corresponding experiments: the variation in the static E-field strength within the interaction range between the molecule and beams 1 and 2 should be much smaller than 1 kV/cm. On the other hand, as shown in Fig. 3 of the main text, the enantiospecific transition can be completed with a typical molecule-beam interaction time of about 0.5 μs. At room temperature (300 K), a 1,2-propanediol molecule (the molecule of Fig. 2) can travel approximately 0.16 mm during this time. Thus, a molecule-beam interaction length scale of 0.5 mm to 1 mm would be sufficient. At this scale, the spatial fluctuation of the static E-field strength should be much smaller than 1 kV/cm.

S8 Experimental Feasibility of Observing Enantiospecific Transition Effects

S8.1 Experimental Approaches

In our main text we show two types of effects induced by the enantiospecific transition, i.e.,

(1) Enantiospecific population transfer (ESPT), which are shown in Fig. 3(A-C).

(2) Enantiospecific absorption (ESA) or enantiospecific fluorescence (or microwave) emission of molecules excited to the γ-state. A example of ESA is shown in Fig. 3D.

Both of these two types of effects may be observed in experiments. Specifically, ESPT can be observed by measuring the population of molecules in the state $|\gamma, 0, s\rangle$ ($s = L, R$) in experiments sample of molecules s. This population is proportional to $P_{\gamma}^{(s)}$ and can be determined, for example, by further exciting the molecules in the state $|\gamma, 0, s\rangle$ to a higher electronic state and detecting the resulting fluorescence (*40, 43*).

Additionally, ESA can be observed by measuring the absorption rate A of beam 2. As defined in the caption of Fig. 3 in the main text, this rate is determined from the ratio of the intensities of beam 2 before and after passing through the molecular sample. Furthermore, enantiospecific fluorescence (or microwave) emission can be directly observed by measuring the intensity of the emitted fluorescence or microwaves.

Moreover, the ESA shown in Fig. 3D corresponds to the case where beam 2 is weak. Actually, ESA or enantiospecific fluorescence (or microwave) emission can also occur when beam 2 is strong. In this case, to observe a significant variation in the absorption rate or fluorescence (or microwave) intensity with the polarization angle θ of beam 2, in the presence of the static electric field, one can first allow the molecules to interact only with beam 1 for a while, then switch off beam 1 and apply beam 2, followed by performing the measurements.

S8.2 Research Contents of the Following Subsections

In this section, we examine the impacts of errors in the experimental control parameters, as well as the thermal population and intermolecular collisions, on the above effects induced by the

enantiospecific transition. Specifically, we consider the examples of ESPT with the initial state α and ESA for beam 2 in systems with $\epsilon_{\alpha,0} < \epsilon_{\beta,\pm 1} < \epsilon_{\gamma,0}$, as shown in Fig. 3(A and D) of the main text, focusing on the cases where the polarization angle θ of beam 2 is set to $\theta_{\mathrm{f}}^{(L)}$. The impacts of above issues on enantiospecific fluorescence (or microwave) emission are similar as those on ESA.

As demonstrated in the main text, in the above examples the two-photon transition $\alpha \longrightarrow \gamma$ is forbidden for the L-handed molecule, while it is allowed for the R-handed molecule. Consequently, in the ideal case, the time-averaged final-state probability $\bar{P}_{\gamma}^{(s)}$ ($s = L, R$) of a molecule with chirality s in ESPT, and the absorption rate $A^{(s)}$ of beam 2 in ESA for a pure ensemble of molecules with chirality s, satisfy $\bar{P}_{\gamma}^{(L)} = A^{(L)} = 0$, whereas $\bar{P}_{\gamma}^{(R)} \neq 0$ and $A^{(R)} \neq 0$. As noted in the main text, here $s = L/R$ denotes the left- and right-handed chirality.

S8.3 Impacts of Frequency Errors and Random Phase Fluctuations of the Beams.

In realistic experiments the phases of beams 1 and 2 have frequency errors and random fluctuations. In the presence of them, the complex amplitude of beam j ($j = 1, 2$) should be revised from $\mathcal{E}_j e^{-i\omega_j t}$ to $\mathcal{E}_j e^{-i\omega_j t - i\delta\omega_j t + i\phi_j(t)}$. Here $\delta\omega_j$ and $\phi_j(t)$ ($j = 1, 2$) are the frequency error and random phase fluctuation of the beam j, respectively.

To investigate the impact of $\delta\omega_j$ and $\phi_j(t)$ ($j = 1, 2$), we substitute the updated complex amplitudes $\mathcal{E}_j e^{-i\omega_j t - i\delta\omega_j t + i\phi_j(t)}$ ($j = 1, 2$) into the calculations presented in Sec. S5. As a result of this modification, an additional term

$$\delta\hat{H}^{(s)} := \left(\dot{\phi}_1(t) - \delta\omega_1\right) \sum_{M=\pm 1} |\beta, M; s\rangle\langle\beta, M; s| + \left(\dot{\phi}_1(t) + \dot{\phi}_2(t) - \delta\omega_1 - \delta\omega_2\right) |\gamma, 0; s\rangle\langle\gamma, 0; s|, \ (s = L, R) \tag{S52}$$

must be added to the expression of the molecular Hamiltonian $\hat{H}^{(s)}$ in the rotating frame, i.e., the right-hand side of Eq. (S27). Here, $\dot{\phi}_{1(2)}(t)$ denotes the time derivative of $\phi_{1(2)}(t)$. Note that $\dot{\phi}_{1(2)}(t)$ are stochastic processes that satisfy the following statistical relationships: $\langle\dot{\phi}_1(t)\rangle = \langle\dot{\phi}_2(t)\rangle = \langle\dot{\phi}_1(t)\dot{\phi}_2(t')\rangle = 0$, $\langle\dot{\phi}_1(t)\dot{\phi}_1(t')\rangle = \Gamma_1\delta(t - t')$, and $\langle\dot{\phi}_2(t)\dot{\phi}_2(t')\rangle = \Gamma_2\delta(t - t')$, where $\langle\ldots\rangle$ denotes the sample mean for the stochastic process (*62*), and $\Gamma_{1(2)}$ represents the amplitude of the random phase fluctuation of beam 1 (2).

Furthermore, since the term $\delta\hat{H}^{(s)}$ does not induce transitions among the α-, β-, and γ-states, it cannot alter the TPSR of our system or shift the values of $\theta_{\rm f}^{(L,R)}$. Therefore, when $\theta = \theta_{\rm f}^{(L)}$, the two-photon transition for molecule L (R) is always forbidden (permitted) by the TPSR, even in the presence of frequency errors $\delta\omega_{1,2}$ and random phase fluctuations $\phi_{1,2}$. As a result, $\delta\omega_{1,2}$ and $\phi_{1,2}$ do not change the facts that $\bar{P}_{\gamma}^{(L)} = A^{(L)} = 0$ and $\bar{P}_{\gamma}^{(R)} \neq 0$, $A^{(R)} \neq 0$. At most, they can qualitatively affect the non-zero transition probability $\bar{P}_{\gamma}^{(R)}$ or the non-zero absorption rate $A^{(R)}$ for molecule R. We illustrate this result in Fig. S6, where $\bar{P}_{\gamma}^{(L,R)}$ and $A^{(L,R)}$ are illustrated as functions of the frequency errors $\delta\omega_{1,2}$ and the random phase fluctuation amplitude $\Gamma_{1,2}$, for the cases in Fig. 3(A and D) with $\theta = \theta_{\rm f}^{(L)}$.

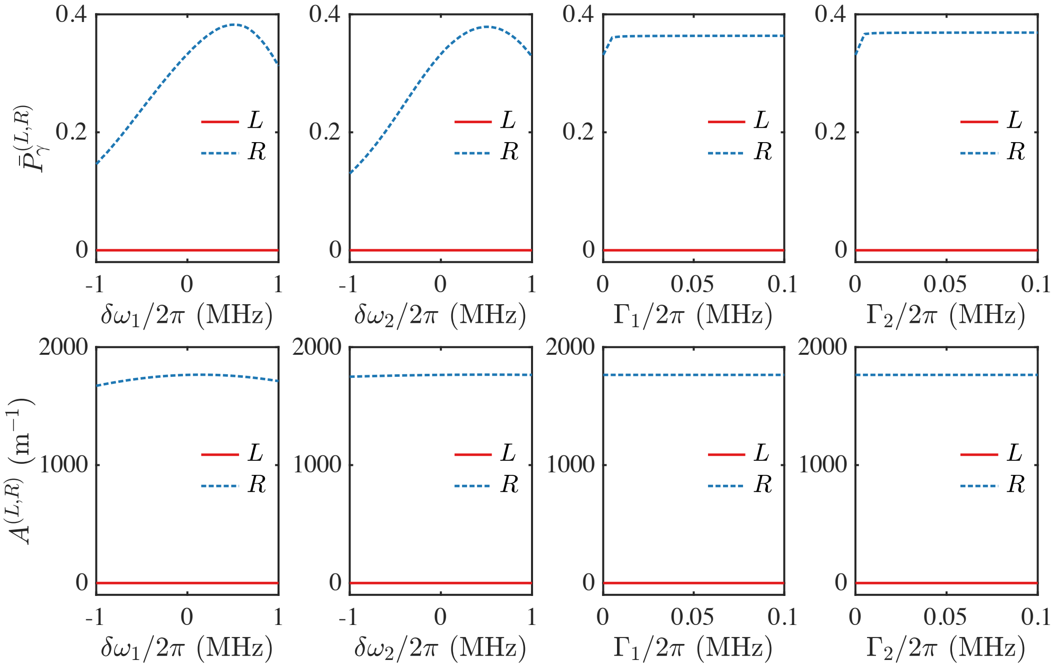

Figure S6: **Probability $\bar{P}_{\gamma}^{(L,R)}$ and absorption rate $A^{(L,R)}$ as functions of frequency errors and random phase fluctuations. Top row:** $\bar{P}_{\gamma}^{(L,R)}$ as functions of $\delta\omega_{1,2}$ and $\Gamma_{1,2}$, for the cases in Fig. 3A. **Bottom row:** $A^{(L,R)}$ as functions of $\delta\omega_{1,2}$ and $\Gamma_{1,2}$, for the cases in Fig. 3D with $\theta = \theta_{\rm f}^{(L)}$. In each figure, one of the four parameters $(\delta\omega_1, \delta\omega_2, \Gamma_1, \Gamma_2)$ acts as the x-label, and the other three are all set to zero.

In the end of this part, we discuss the impact of the parity-violating energy difference between the enantiomers, which is estimated to be less than $(2\pi)1$ Hz (*63*). The presence of this energy difference

is equivalent to a tiny difference between the detunings $\Delta_1 = \epsilon_{\beta,1} - \epsilon_{\alpha,0} - \omega_1$, $\Delta_2 = \epsilon_{\gamma,0} - \epsilon_{\beta,1} - \omega_2$ experienced by molecules L and those experienced by molecule R. Using an analysis which is similar as above, we find that the impact of this energy difference is the same as the ones of the error in beam frequencies. Specifically, it can neither alter the TPSR and the values of $\theta_{\mathrm{f}}^{(L,R)}$, nor qualitatively change the effects of ESPT and ESA. This energy difference can only induce quantitative effects on the molecule for which the two-photon cascade transition is permitted. Furthermore, since the parity-violating energy difference is much weaker than other characteristic energies in our system (such as the beam-molecule coupling strength $\Omega_{1,2}$), these induced quantitative effects are negligible. Thus, in this work we totally ignore this energy difference.

S8.4 Impacts of Errors in the Polarization Angle θ of Beam 2.

As shown in our main text, beam 2 is polarized along $\mathbf{e}_2 = \sin(\theta/2)\mathbf{e}_x - \cos(\theta/2)\mathbf{e}_y$. If there is error in θ in a realistic experiment, the results of ESPT and ESA may undergo unexpected changes. Nevertheless, as shown in Fig. 3(C and D), these results are quite robust with respect to θ. Thus, qualitatively speaking, if the error of θ (denoted as $\delta\theta$) is much less than one radian, the induced variation of the results of ESPT and ESA are negligible.

To quantitatively investigate the influence of the error $\delta\theta$ on ESPT and ESA, we introduce the following two visibilities:

$$v_{\mathrm{ESPT}} = \left| \frac{\bar{P}_{\gamma}^{(R)} - \bar{P}_{\gamma}^{(L)}}{\bar{P}_{\gamma}^{(R)} + \bar{P}_{\gamma}^{(L)}} \right|; \qquad v_{\mathrm{ESA}} = \left| \frac{A^{(R)} - A^{(L)}}{A^{(R)} + A^{(L)}} \right|. \tag{S53}$$

Note that v_{ESPT} is enantiomeric excess of the γ-state (*3*). It is evident that when θ is set to $\theta_{\mathrm{f}}^{(L)}$, in the ideal cases we have $v_{\mathrm{ESPT}} = v_{\mathrm{ESA}} = 1$, due to the facts that $\bar{P}_{\gamma}^{(L)} = A^{(L)} = 0$ and $\bar{P}_{\gamma}^{(R)} \neq 0$, $A^{(R)} \neq 0$. In the presence of errors, we find that $v_{\mathrm{ESPT,ESA}} < 1$. Therefore, the difference between $v_{\mathrm{ESPT(ESA)}}$ and unity describes the influence of the errors on ESPT (ESA).

We consider the systems depicted in Fig. 3(A and D), with θ set to $\theta_{\mathrm{f}}^{(L)}$. In the presence of the error $\delta\theta$, we have $\theta = \theta_{\mathrm{f}}^{(L)} + \delta\theta$. In Fig. S7, we show the visibilities v_{ESPT} and v_{ESA} for these systems. It is observed that, for $|\delta\theta| \ll 1$, the deviation of these visibilities from 1, which reflects the impact of the error, is negligible. That is consistent with the above qualitative analysis. Additionally, the condition $|\delta\theta| \ll 1$ is achievable in current experiments.

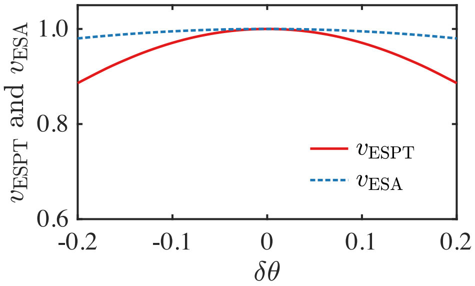

Figure S7: **Impacts of errors in the polarization angle θ of beam 2.** Here we show the visibilities v_{ESPT} (red solid line) and v_{ESA} (blue dotted line), for the system of Fig. 3A and Fig. 3D with $\theta = \theta_{\mathrm{f}}^{(L)} = \pi/2$, respectively, as functions of the errors $\delta\theta$.

Finally, we would like to state an issue related to polarization adjustment. In current experiments, the polarization direction of lasers can be easily modified using devices such as polarizers. However, for microwaves, once the polarization direction is fixed, it is generally more difficult to change. Therefore, if it is necessary to measure the variation of certain physical quantities (such as the absorption of beam 2 or the occupation of the γ-state) with respect to the polarization angle θ of beam 2, it is preferable to choose a laser rather than a microwave for beam 2. Of course, the experiments with θ being fixed can be performed with beam 2 as either a laser or a microwave.

S8.5 Impacts of Thermal and Collisional effects.

Now, we discuss the influence of thermal occupation of the β- and γ-states, as well as intermolecular collisions. In the presence of these two issues, the quantum coherence of the system—particularly the coherence between the two β-states, which plays a key role in the occurrence of TPSR—can be disrupted. Furthermore, additional transition processes to the γ-state, which are permitted for both molecules L and R, may be induced. As a result, the effects of ESPT and ESA may be weakened.

Therefore, in experiments of ESPT or ESA one should try to avoid the above issues, or suppress their negative impact. Generally speaking, this can be achieved via the following two approaches:

(1) Choosing the β- and γ-states to have sufficiently high energies so that thermal occupation of these states is negligible, so that the energies of the β- and γ-states are significantly greater than the thermal energy, which ranges from approximately 14 cm^{-1} to 209 cm^{-1} for typical

temperatures between 20 and 300 K. This can be done, for example, by selecting these states in high vibrational or electronic levels. Notice that as mentioned in the main text, a key advantage of the method proposed in this work for manipulating chiral molecules is the flexibility in selecting the relevant states.

(2) Performing the experiment in a system with low or negligible collisional rates, such as low-pressure molecular gases with low number density, or molecule beams, as in the experiment described in (*40, 42, 43*).

Additionally, in the following we propose two specific approaches for ESPT and ESA, respectively, which can suppress of the negative impacts of thermal population or intermolecular collisions.

(A) ESPT.

For ESPT, we propose an approach to mitigate the adverse effect of thermal occupation in the β-states. This approach is particularly relevant for systems in which thermal occupation of the γ-states can be safely neglected, whereas that of the β-states remains significant.

Our approach involves tuning the beam frequencies to simultaneously satisfy the one-photon large-detuning condition ($|\Delta_{1(2)}| \gg \Omega_{1(2)}$) and the two-photon near-resonance condition ($|\Delta_1+\Delta_2| \approx (0.1-0.2)\Omega_{1,2}$), where $\Delta_1 = \epsilon_{\beta,1} - \epsilon_{\alpha,0} - \omega_1$ and $\Delta_2 = \epsilon_{\gamma,0} - \epsilon_{\beta,1} - \omega_2$, with $\Omega_{1(2)}$ being the Rabi frequency of beam 1 (2), as defined previously. In this regime, the two-photon transition $\alpha \longrightarrow \gamma$ proceeds with negligible instantaneous population of the β-states, and thus the ESPT effect remains essentially unaffected by their thermal occupation.

To illustrate this approach, we calculate the visibility v_{ESPT} for the process shown in Fig. 3A of the main text, with mixed initial state

$$\hat{\rho}^{(s)} = (1-p_\beta)|\alpha,0;s\rangle\langle\alpha,0;s| + \frac{p_\beta}{2}\left(|\beta,+1;s\rangle\langle\beta,+1;s| + |\beta,-1;s\rangle\langle\beta,-1;s|\right) \qquad \text{(S54)}$$

for molecule $s = L, R$. Here, p_β represents the initial thermal population of the β-level. As shown in Fig. S8, with the original parameters of Fig. 3A, v_{ESPT} decreases rapidly as p_β increases. By contrast, when the beam frequencies are tuned to $|\Delta_1| = 10\Omega_{1,2}$, $|\Delta_1+\Delta_2| = 0.2\Omega_{1,2}$, so that the above two conditions are satisfied, the visibility decays much slower with p_β, and remains nearly

0.9 even when p_β reaches 0.5. It is worth noting that, in the case of complete two-photon resonance (i.e., $\Delta_1 + \Delta_2$ is exactly zero), the sensitivity of the $v_{\rm ESPT}$ to θ can potentially increase.

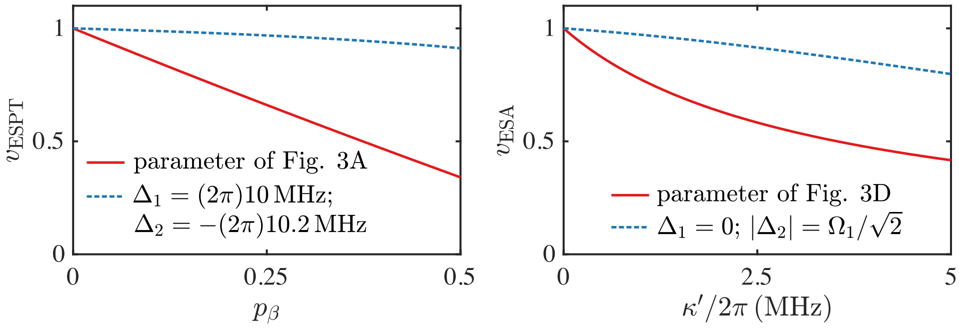

Figure S8: **Effects of approaches for mitigating thermal and collisional impacts. Left:** Visibility $v_{\rm ESPT}$ for the ESPT process of Fig. 3A of the main text, with mixed initial state of Eq. (S54). Here we show the results with the original parameters of Fig. 3A (red solid line), and the ones with beam frequencies being tuned to $\Delta_1 = (2\pi)10\,$MHz and $\Delta_2 = -(2\pi)10.2\,$MHz, so that the conditions $|\Delta_{1(2)}| \gg \Omega_{1(2)}$ and two-photon near-resonance are satisfied, and other parameters being the same as the ones of Fig. 3A (blue dotted line). **Right**: Visibility $v_{\rm ESA}$ for the ESA process of Fig. 3D of the main text with $\theta = \theta_{\rm f}^{(L)} = \pi/2$, as a function of κ'. Here we show the results with the original parameters of Fig. 3D (red solid line), and the ones with $\Delta_1 = 0$ and $|\Delta_2| = \Omega_1/\sqrt{2}$, $\Omega_1 = (2\pi)150\,$MHz, and other parameters being the same as the ones of Fig. 3D (blue dotted line).

(B) ESA.

For ESA, we propose an approach to mitigate the negative influence of decoherence induced by intermolecular collisions. We first introduce the approach and illustrate its effects, followed by an explanation of its underlying principle.

Our approach involves tuning the frequencies of beams 1 and 2, along with the intensity of beam 1, so that the detunings $\Delta_{1,2}$ and the Rabi frequency Ω_1 satisfy $\Delta_1 = 0$ and $|\Delta_2| = \Omega_1/\sqrt{2} \gg \kappa$.

To illustrate the effect of our approach, we calculate the visibility $v_{\rm ESA}$ for the case of Fig. 3D with $\theta = \theta_{\rm f}^{(L)}$. In our calculation we modify Eq. (S45) by adding a pure dephasing term $\kappa'\{\mathcal{L}[\sigma^z_{\beta+,\gamma;s}]\hat{\rho}^{(s)}(t)+\mathcal{L}[\sigma^z_{\beta-,\gamma;s}]\hat{\rho}^{(s)}(t)+\mathcal{L}[\sigma^z_{\alpha,\gamma;s}]\hat{\rho}^{(s)}(t)+\mathcal{L}[\sigma^z_{\alpha,\beta+;s}]\hat{\rho}^{(s)}(t)+\mathcal{L}[\sigma^z_{\alpha,\beta-;s}]\hat{\rho}^{(s)}(t)+$

$\mathcal{L}[\sigma^z_{\beta-,\beta+;s}]\hat{\rho}^{(s)}(t)\}$ on the right-hand side, which phenomenologically describes the molecular collision processes. Here κ' is the collisional relaxation rate and the operator $\sigma^z_{o_1,o_2;s}$ is defined by $\sigma^z_{o_1,o_2;s} = |\overline{o_2;s}\rangle\langle\overline{o_2;s}| - |\overline{o_1;s}\rangle\langle\overline{o_1;s}|$, e.g. $\sigma^z_{\beta+,\gamma;s} = |\overline{\gamma,0;s}\rangle\langle\overline{\gamma,0;s}| - |\overline{\beta,+1;s}\rangle\langle\overline{\beta,+1;s}|$. As shown in the right panel of Fig. S8, when the beam frequencies and the intensity of beam 1 are tuned to meet the aforementioned conditions, the decay of the visibility v_{ESA} with the decoherence rate κ' becomes significantly slower compared to the case with the original parameters shown in Fig. 3D.

Next, we introduce the principle of our approach. Among the collision-induced decoherence effects, the destruction of coherence between the states $|\beta,+1;s\rangle$ and $|\beta,-1;s\rangle$ (with $s = L, R$) has the most detrimental effect on ESA. As shown in the main text, beam 1 couples only the state $|\alpha,0;s\rangle$ to the dressed state $|\psi;s\rangle$ defined in Eq. (3): $|\psi;s\rangle = \mathcal{N}_s^{-1}\left[a_-^{(s)}|\beta,-1;s\rangle + a_+^{(s)}|\beta,+1;s\rangle\right]$. In the ideal case, all molecules in the β-states are in the state $|\psi;s\rangle$, while the other dressed state $|\bar{\psi};s\rangle = \mathcal{N}_s^{-1}\left[a_+^{(s)*}|\beta,-1;s\rangle - a_-^{(s)*}|\beta,+1;s\rangle\right]$, orthogonal to $|\psi;s\rangle$, is not occupied. This coherence is crucial for the formation of TPSR. However, if the coherence between $|\beta,+1;s\rangle$ and $|\beta,-1;s\rangle$ is lost, the molecule will be in a mixed state, with a non-zero probability of occupying $|\bar{\psi};s\rangle$. In this case, the ESA effect, resulting from the TPSR, would be nullified.

In our approach, when the molecule s is in the state $|\bar{\psi};s\rangle$, it absorbs almost none of beam 2 due to the large detuning $|\Delta_2| \gg \kappa$. On the other hand, thanks to beam 1 and the condition $\Delta_1 = 0$, the states $|\psi;s\rangle$ and $|\alpha,0;s\rangle$ form two dressed states $(|\psi;s\rangle \pm |\alpha,0;s\rangle)/\sqrt{2}$, with energies $\pm\Omega_1/\sqrt{2}$ in the rotating frame. The condition $|\Delta_2| = \Omega_1/\sqrt{2}$ ensures that beam 2 is resonant with the transition between one of the dressed states and the γ-state. As a result, molecules in the state $|\psi;s\rangle$ can still strongly absorb beam 2. To summarize, under these conditions, the absorption of beam 2 is primarily contributed by molecules in the state $|\psi;s\rangle$, meaning the collision-induced occupation of $|\bar{\psi};s\rangle$ has little effect on the variation of absorption with the polarization angle θ of beam 2.

REFERENCES AND NOTES

1. P. G. Mezey, *New Developments in Molecular Chirality* (Springer, Netherlands, 1991).

2. D. Patterson, M. Schnell, J. M. Doyle, Enantiomer-specific detection of chiral molecules via microwave spectroscopy. *Nature* **497**, 475–477 (2013).

3. D. Patterson, J. M. Doyle, Sensitive chiral analysis via microwave three-wave mixing. *Phys. Rev. Lett.* **111**, 023008 (2013).

4. D. Patterson, M. Schnell, New studies on molecular chirality in the gas phase: Enantiomer differentiation and determination of enantiomeric excess. *Phys. Chem. Chem. Phys.* **16**, 11114–11123 (2014).

5. S. Lobsiger, C. Perez, L. Evangelisti, K. K. Lehmann, B. H. Pate, Molecular structure and chirality detection by Fourier transform microwave spectroscopy. *J. Phys. Chem. Lett.* **6**, 196–200 (2015).

6. V. A. Shubert, D. Schmitz, C. Pérez, C. Medcraft, A. Krin, S. R. Domingos, D. Patterson, M. Schnell, Chiral analysis using broadband rotational spectroscopy. *J. Phys. Chem. Lett.* **7**, 341–350 (2016).

7. A. Yachmenev, S. N. Yurchenko, Detecting chirality in molecules by linearly polarized laser fields. *Phys. Rev. Lett.* **117**, 033001 (2016).

8. K. K. Lehmann, "Theory of enantiomer-specific microwave spectroscopy" in *Frontiers and Advances in Molecular Spectroscopy* (Elsevier, 2018).

9. K. K. Lehmann, Influence of spatial degeneracy on rotational spectroscopy: Three-wave mixing and enantiomeric state separation of chiral molecules. *J. Chem. Phys.* **149**, 094201 (2018).

10. C. Ye, Q. Zhang, Y.-Y. Chen, Y. Li, Determination of enantiomeric excess with chirality-dependent ac Stark effects in cyclic three-level models. *Phys. Rev. A* **100**, 033411 (2019).

11. C. Ye, Y. Sun, X. Zhang, Entanglement-assisted quantum chiral spectroscopy. *J. Phys. Chem. Lett.* **12**, 8591–8597 (2021).

12. M.-R. Cai, C. Ye, H. Dong, Y. Li, Enantiodetection of chiral molecules via two-dimensional spectroscopy. *Phys. Rev. Lett.* **129**, 103201 (2022).

13. Y.-Y. Chen, J.-J. Cheng, C. Ye, Y. Li, Enantiodetection of cyclic three-level chiral molecules in a driven cavity. *Phys. Rev. Res.* **4**, 013100 (2022).

14. Y.-H. Kang, Z.-P. Lin, J.-Q. Yang, J. Song, Y. Xia, Near-perfect discrimination of chiral molecules based on steady states of a cavity mode. *Phys. Rev. A* **107**, 053714 (2023).

15. C. Ye, Y. Sun, Y. Li, X. Zhang, Single-shot nondestructive quantum sensing for gaseous samples with hundreds of chiral molecules. *J. Phys. Chem. Lett.* **14**, 6772–6777 (2023).

16. Y. Ke, Z. Song, Q.-D. Jiang, Vacuum-induced symmetry breaking of chiral enantiomer formation in chemical reactions. *Phys. Rev. Lett.* **131**, 223601 (2023).

17. Y. Li, C. Bruder, C. P. Sun, Generalized Stern-Gerlach effect for chiral molecules. *Phys. Rev. Lett.* **99**, 130403 (2007).

18. X. Li, M. Shapiro, Theory of the optical spatial separation of racemic mixtures of chiral molecules. *J. Chem. Phys.* **132**, 194315 (2010).

19. A. Eilam, M. Shapiro, Spatial separation of dimers of chiral molecules. *Phys. Rev. Lett.* **110**, 213004 (2013).

20. B. Liu, C. Ye, C. P. Sun, Y. Li, Spatial enantioseparation of gaseous chiral molecules. *Phys. Rev. A* **104**, 013113 (2021).

21. P. Král, M. Shapiro, Cyclic population transfer in quantum systems with broken symmetry. *Phys. Rev. Lett.* **87**, 183002 (2001).

22. P. Král, I. Thanopulos, M. Shapiro, D. Cohen, Two-step enantio-selective optical switch. *Phys. Rev. Lett.* **90**, 033001 (2003).

23. Y. Li, C. Bruder, Dynamic method to distinguish between left- and right-handed chiral molecules. *Phys. Rev. A* **77**, 015403 (2008).

24. W. Z. Jia, L. F. Wei, Distinguishing left- and right-handed molecules using two-step coherent pulses. *J. Phys. B At. Mol. Opt. Phys.* **43**, 185402 (2010).

25. M. Leibscher, T. F. Giesen, C. P. Koch, Principles of enantio-selective excitation in three-wave mixing spectroscopy of chiral molecules. *J. Chem. Phys.* **151**, 014302 (2019).

26. N. V. Vitanov, M. Drewsen, Highly efficient detection and separation of chiral molecules through shortcuts to adiabaticity. *Phys. Rev. Lett.* **122**, 173202 (2019).

27. C. Ye, Q. Zhang, Y.-Y. Chen, Y. Li, Effective two-level models for highly efficient inner-state enantioseparation based on cyclic three-level systems of chiral molecules. *Phys. Rev. A* **100**, 043403 (2019).

28. B. T. Torosov, M. Drewsen, N. V. Vitanov, Chiral resolution by composite Raman pulses. *Phys. Rev. Res.* **2**, 043235 (2020).

29. B. T. Torosov, M. Drewsen, N. V. Vitanov, Efficient and robust chiral resolution by composite pulses. *Phys. Rev. A* **101**, 063401 (2020).

30. J.-L. Wu, Y. Wang, J. Song, Y. Xia, S.-L. Su, Y.-Y. Jiang, Robust and highly efficient discrimination of chiral molecules through three-mode parallel paths. *Phys. Rev. A* **100**, 043413 (2019).

31. J.-L. Wu, Y. Wang, J.-X. Han, C. Wang, S.-L. Su, Y. Xia, Y. Jiang, J. Song, Two-path interference for enantiomer-selective state transfer of chiral molecules. *Phys. Rev. Applied* **13**, 044021 (2020).

32. Y. Guo, X. Gong, S. Ma, C.-C. Shu, Cyclic three-level-pulse-area theorem for enantioselective state transfer of chiral molecules. *Phys. Rev. A* **105**, 013102 (2022).

33. M. Leibscher, E. Pozzoli, C. Pérez, M. Schnell, M. Sigalotti, U. Boscain, C. P. Koch, Full quantum control of enantiomer-selective state transfer in chiral molecules despite degeneracy. *Commun. Phys.* **5**, 110 (2022).

34. B. Liu, C. Ye, C. P. Sun, Y. Li, Enantiospecific state transfer for gaseous symmetric-top chiral molecules. *Phys. Rev. A* **105**, 043110 (2022).

35. J.-J. Cheng, Y.-Y. Chen, Y. Li, L. Zhang, Enantiomer-specific state transfer of chiral molecules in cyclic three-level systems with SU(2) structures. *Phys. Rev. A* **107**, 013718 (2023).

36. F. Zou, Y. Li, P. Zhang, Enantiomer-specific pumping of chiral molecules. *J. Phys. Chem. Lett.* **15**, 10554–10559 (2024).

37. S. Eibenberger, J. Doyle, D. Patterson, Enantiomer-specific state transfer of chiral molecules. *Phys. Rev. Lett.* **118**, 123002 (2017).

38. C. Pérez, A. L. Steber, S. R. Domingos, A. Krin, D. Schmitz, M. Schnell, Coherent enantiomer-selective population enrichment using tailored microwave fields. *Angew. Chem. Int. Ed. Engl.* **56**, 12512–12517 (2017).

39. C. Pérez, A. L. Steber, A. Krin, M. Schnell, State-specific enrichment of chiral conformers with microwave spectroscopy. *J. Phys. Chem. Lett.* **9**, 4539–4543 (2018).

40. J. Lee, J. Bischoff, A. O. Hernandez-Castillo, B. Sartakov, G. Meijer, S. Eibenberger-Arias, Quantitative study of enantiomer-specific state transfer. *Phys. Rev. Lett.* **128**, 173001 (2022).

41. W. Sun, D. S. Tikhonov, H. Singh, A. L. Steber, C. Pérez, M. Schnell, Inducing transient enantiomeric excess in a molecular quantum racemic mixture with microwave fields. *Nat. Commun.* **14**, 934 (2023).

42. J. Lee, J. Bischoff, A. O. Hernandez-Castillo, E. Abdiha, B. G. Sartakov, G. Meijer, S. Eibenberger-Arias, The influence of microwave pulse conditions on enantiomer-specific state transfer. *New J. Phys.* **26**, 033015 (2024).

43. J. Lee, E. Abdiha, B. G. Sartakov, G. Meijer, S. Eibenberger-Arias, Near-complete chiral selection in rotational quantum states. *Nat. Commun.* **15**, 7441 (2024).

44. P. J. Stephens, Theory of vibrational circular dichroism. *J. Phys. Chem.* **89**, 748–752 (1985).

45. S. Beaulieu, A. Comby, D. Descamps, B. Fabre, G. A. Garcia, R. Géneaux, A. G. Harvey, F. Légaré, Z. Mašín, L. Nahon, A. F. Ordonez, S. Petit, B. Pons, Y. Mairesse, O. Smirnova, V. Blanchet, Photoexcitation circular dichroism in chiral molecules. *Nat. Phys.* **14**, 484–489 (2018).

46. Y. He, B. Wang, R. K. Dukor, L. A. Nafie, Determination of absolute configuration of chiral molecules using vibrational optical activity: A review. *Appl. Spectrosc.* **65**, 699–723 (2011).

47. M. M. R. Fanood, N. B. Ram, C. S. Lehmann, I. Powis, M. H. M. Janssen, Enantiomer-specific analysis of multi-component mixtures by correlated electron imaging-ion mass spectrometry. *Nat. Commun.* **6**, 7511 (2015).

48. R. Liang, E. Pozzoli, M. Leibscher, M. Sigalotti, C. P. Koch, U. Boscain, Enhancing the controllability of quantum systems via a static field. arXiv:2504.17303 (2025).

49. D. S. Tikhonov, A. Blech, M. Leibscher, L. Greenman, M. Schnell, C. P. Koch, Pump-probe spectroscopy of chiral vibrational dynamics. *Sci. Adv.* **8**, eade0311 (2022).

50. M. Leibscher, E. Pozzoli, A. Blech, M. Sigalotti, U. Boscain, C. P. Koch, Quantum control of rovibrational dynamics and application to light-induced molecular chirality. *Phys. Rev. A* **109**, 012810 (2024).

51. A. D. Buckingham, P. Fischer, Linear electro-optic effect in optically active liquids. *Chem. Phys. Lett.* **297**, 239–246 (1998).

52. P. Fischer, A. D. Buckingham, K. Beckwitt, D. S. Wiersma, F. W. Wise, New electro-optic effect: Sum-frequency generation from optically active liquids in the presence of a dc electric field. *Phys. Rev. Lett.* **91**, 173901 (2003).

53. M. Quack, J. Stohner, M. Willeke, High-resolution spectroscopic studies and theory of parity violation in chiral molecules. *Annu. Rev. Phys. Chem.* **59**, 741–769 (2008).

54. R. N. Zare, *Angular Momentum* (Wiley, New York, 1998).

55. F. J. Lovas, D. F. Plusquellic, B. H. Pate, J. L. Neill, M. T. Muckle, A. J. Remijan, Microwave spectrum of 1,2-propanediol. *J. Mol. Spectrosc.* **257**, 82–93 (2009).

56. A. Jacob, K. Hornberger, Effect of molecular rotation on enantioseparation. *J. Chem. Phys.* **137**, 044313 (2012).

57. C. Ye, Q. Zhang, Y. Li, Real single-loop cyclic three-level configuration of chiral molecules. *Phys. Rev. A* **98**, 063401 (2018).

58. B. E. Arenas, "High-resolution broadband rotational spectroscopy and electrical discharge experiments of astrochemically relevant molecules," Ph.D. thesis, Universität Hamburg, Germany (2020); https://ediss.sub.uni-hamburg.de/handle/ediss/8742.

59. J. J. Sakurai, J. Napolitano, *Modern Quantum Mechanics* (Cambridge Univ. Press, 2000).

60. M. O. Scully, M. S. Zubairy, *Quantum Optics* (Cambridge Univ. Press, 2012).

61. D. T. Moore, L. Oudejans, R. E. Miller, Pendular state spectroscopy of an asymmetric top: Parallel and perpendicular bands of acetylene-HF. *J. Chem. Phys.* **110**, 197–208 (1999).

62. L. Diósi, Laser linewidth hazard in optomechanical cooling. *Phys. Rev. A* **78**, 021801 (2008).

63. S. K. Tokunaga, C. Stoeffler, F. Auguste, A. Shelkovnikov, C. Daussy, A. Amy-Klein, C. Chardonnet, B. Darquié, Probing weak force-induced parity violation by high-resolution mid-infrared molecular spectroscopy. *Mol. Phys.* **111**, 2363–2373 (2013).
